# Receptor Activation of HIV-1 Env Leads to Asymmetric Exposure of the gp41 Trimer

**DOI:** 10.1371/journal.ppat.1006098

**Published:** 2016-12-19

**Authors:** Mukta D. Khasnis, Konstantine Halkidis, Anshul Bhardwaj, Michael J. Root

**Affiliations:** Department of Biochemistry and Molecular Biology, Sidney Kimmel Cancer Center, Thomas Jefferson University, Philadelphia, PA, United States of America; University of Zurich, SWITZERLAND

## Abstract

Structural rearrangements of HIV-1 glycoprotein Env promote viral entry through membrane fusion. Env is a symmetric homotrimer with each protomer composed of surface subunit gp120 and transmembrane subunit gp41. Cellular CD4- and chemokine receptor-binding to gp120 coordinate conformational changes in gp41, first to an extended prehairpin intermediate (PHI) and, ultimately, into a fusogenic trimer-of-hairpins (TOH). HIV-1 fusion inhibitors target gp41 in the PHI and block TOH formation. To characterize structural transformations into and through the PHI, we employed asymmetric Env trimers containing both high and low affinity binding sites for individual fusion inhibitors. Asymmetry was achieved using engineered Env heterotrimers composed of protomers deficient in either CD4- or chemokine receptor-binding. Linking receptor engagement to inhibitor affinity allowed us to assess conformational changes of individual Env protomers in the context of a functioning trimer. We found that the transition into the PHI could occur symmetrically or asymmetrically depending on the stoichiometry of CD4 binding. Sequential engagement of multiple CD4s promoted progressive exposure of individual fusion inhibitor binding sites in a CD4-dependent fashion. By contrast, engagement of only a single CD4 molecule led to a delayed, but symmetric, exposure of the gp41 trimer. This complex coupling between Env-CD4 interaction and gp41 exposure explained the multiphasic fusion-inhibitor titration observed for a mutant Env homotrimer with a naturally asymmetric gp41. Our results suggest that the spatial and temporal exposure of gp41 can proceed in a nonconcerted, asymmetric manner depending on the number of CD4s that engage the Env trimer. The findings have important implications for the mechanism of viral membrane fusion and the development of vaccine candidates designed to elicit neutralizing antibodies targeting gp41 in the PHI.

## Author Summary

For HIV, cellular invasion requires merging viral and cellular membranes, an event achieved through the activity of the viral fusion protein Env. Env consists of three gp120 and three gp41 subunits symmetrically arranged on the viral surface. The gp120 subunits bind cellular receptors, which, in turn, coordinate gp41 conformational changes that promote membrane fusion. Understanding these structural rearrangements illuminates the mechanism of viral membrane fusion, and also spurs development of targeted inhibitors of viral entry and vaccine candidates that elicit antiviral immune responses. In this study, we employed a novel strategy to investigate individual subunits in the context of functioning Env complexes. The strategy links distinct gp120-receptor interactions to conformational changes that expose specific gp41 subunits. We found that, despite the initial symmetric arrangement of its subunits, Env conformational changes most often proceed quite asymmetrically, leading to exposure of only one-third of the gp41 trimer for much of the fusion event. This finding might explain why attempts to elicit potent anti-HIV antibodies to a fully exposed gp41 trimer have been largely unsuccessful. The study gives us a glimpse of the early structural transitions leading to Env-mediated membrane fusion and provides a framework for interrogating the fusion proteins of other membrane-encapsulated viruses.

## Introduction

**Fig 1:**
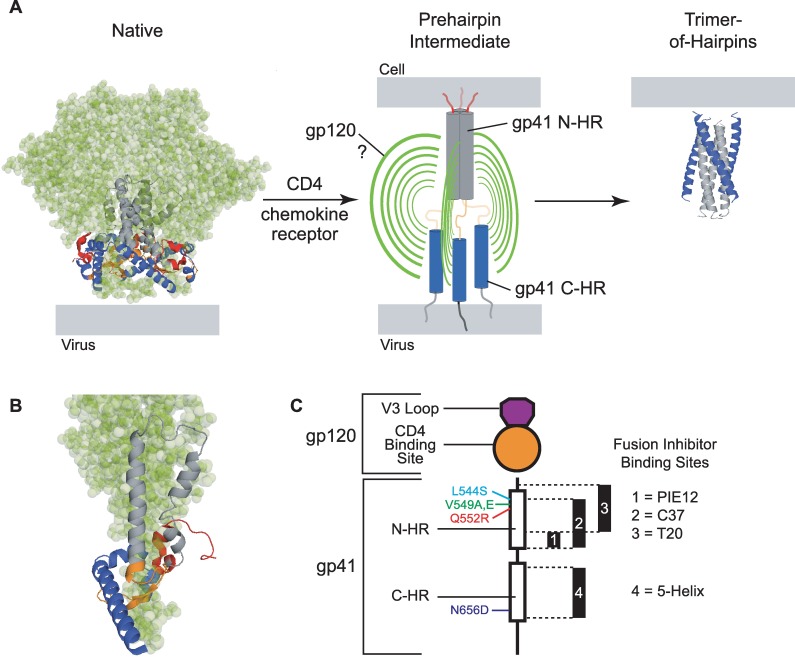
Structural transformations of the HIV-1 Env trimer during viral entry. (**A**) In the native, prefusogenic conformation (PDB ID: 5FYL, [11]), gp41 subunits (ribbon diagram) are held in a metastable conformation by a canopy of gp120 subunits (green space-filling model). In the orientation shown, the viral membrane is at the base of the modeled structure. Cellular receptor binding to gp120 releases constraints on the gp41 N-HR and C-HR segments, enabling the N-HR coiled coil to form and extend the gp41 N-termini toward the target cell membrane. The disposition of gp120 subunits in this transient intermediate state is unknown. Ultimately, the N-HR and C-HR segments collapse into a six-helix bundle (PDB ID: 1AIK, [12]) that stabilizes the postfusogenic gp41 trimer-of-hairpins. In this conformation, the gp41 N-terminal fusion peptide and transmembrane segments (not depicted), as well as their associated membranes, are on the same side of the molecule (top). The fusion inhibitors used in this study target the N-HR coiled coil and C-HR segments transiently exposed during the prehairpin intermediate state. (**B**) Closeup of a single gp41 ectodomain (ribbon diagram) in its metastable native conformation cradled by its cognate gp120 subunit (green). From N- to C-terminus, the regions of the gp41 ectodomain are color coded as follows: fusion peptide and fusion-peptide proximal region—red; N-terminal heptad repeat (N-HR)—grey; disulfide-bonded loop—orange; C-terminal heptad repeat (C-HR)—blue. The membrane proximal-external region (MPER) is not shown. (**C**) Schematic of a single Env protomer highlighting gp120 and gp41 segments modified for this study. Fusion inhibitor binding sites in the N-HR and C-HR regions and the relative positions of escape mutations are indicated. The C37 binding site is targeted by both C37-KYI and di-C37, while the 5-Helix binding site is targeted by both 5H_WT_ and 5H_LAVA_.

Entry of human immunodeficiency virus type 1 (HIV-1) into target cells involves fusion of viral and cellular membranes, a process mediated by the viral surface protein Env (gp160) [1]. This heavily glycosylated, type 1 transmembrane protein assembles as a homotrimer following synthesis in the endoplasmic reticulum of virus-producing cells. In the Golgi apparatus, each protomer is cleaved into two subunits that remain noncovalently associated: an N-terminal surface protein (denoted SU or gp120) and a C-terminal transmembrane protein (denoted TM or gp41). Cryo-EM studies on HIV-1 particles revealed that the Env trimer assumes a lobed, mushroom-like appearance, with the gp120 subunits forming a canopy that surrounds a stalk formed by gp41 [2–5]. In high-resolution structures of the Env ectodomain, the N-terminal portion of each gp41, including a 3,4-hydrophobic heptad repeat sequence denoted the N-HR, is cradled by the conserved interior region of a single gp120 subunit (Fig 1) [6–11]. Parts of the N-HR segments adopt a homotrimeric coiled-coil conformation that stabilizes the trimeric interface. The C-terminal portion of the gp41 ectodomain, including a second heptad repeat (denoted C-HR), interacts with and extends beyond the membrane-proximal face of the gp120 trimer (Fig 1). At the other end of the complex, variable loops (V1/V2 and V3) from each gp120 partake in intersubunit interactions to effectively cap the gp120 canopy [6, 7, 10].

In mediating HIV-1 entry, Env undergoes a series of coordinated structural transformations initiated when gp120 binds cellular CD4 [13–15]. This event substantially alters gp120 structure, releasing constraints on the V3 loop and enabling formation of a new antigenic surface called the bridging sheet [16–18]. The released V3 loop and bridging sheet interact with the extracellular loops and N-terminus of chemokine receptors such as CXCR4 or CCR5 (denoted as the coreceptor) [19–22]. CD4-binding also modifies the interaction between gp120 and gp41, often resulting in irreversible shedding of gp120 [23, 24]. Critical to fusion, the gp41 ectodomain can now extend and insert its N-terminal fusion peptide segment into the target cell membrane, thereby spanning the gap between viral and cellular membranes (Fig 1A) [25–27]. Sometime during this extended intermediate state (denoted the prehairpin intermediate state or PHI), the entire gp41 N-HR adopts a coiled-coil conformation, and both the N-HR and C-HR regions become exposed to the extracellular environment (Fig 1A and 1C) [28, 29]. Ultimately, the extended state collapses into its most stable form, a trimer-of-hairpins (TOH), in which the C-HR regions pack in an antiparallel manner into hydrophobic grooves on the outside of the N-HR coiled coil (Fig 1A) [12, 30–32]. The structure brings the fusion peptides and transmembrane domains of the gp41 trimer, and their associated membranes, into the close proximity required for stable fusion [28].

The molecular mechanisms that guide global structural changes of Env are largely unknown. For instance, it remains unclear how the conformational transitions of the gp41 subunits are allosterically coupled to CD4 and coreceptor binding to gp120. As a first step in unraveling these mechanisms, this study focuses on the nature of the native-to-PHI transition that exposes the N-HR and C-HR regions of the gp41 ectodomain. In the current model, the transition is envisioned as a concerted process, with the three gp120 subunits simultaneously disengaging from the gp41 trimer leading to full (symmetric) exposure of the N-HR coiled coil and three C-HR regions [14, 33]. In support of this model, the structure of the Env trimer in its native conformation suggests that all three gp120 subunits must fully separate from the three gp41 N-terminal regions in order for the fusion peptides to extend and N-HR coiled coil to form (Fig 1B) [6, 7]. Furthermore, there is evidence of substantial cooperativity between protomers, which enables the Env trimer to function without engagement of all receptor-binding sites [34–36]. However, biological process involving homomeric proteins rarely proceed in a concerted manner [37, 38]. It is a formal possibility that the Env trimer transitions through one or more asymmetric intermediates, with partial separation of gp120 from the gp41 trimer leading to piecemeal exposure of the gp41 ectodomain.

Probing initial receptor-induced conformational transitions of Env is complicated by the homotrimeric oligomerization of the glycoprotein. The three identical CD4- and coreceptor-binding sites, the three-fold symmetry of the N-HR coiled coil, and the three identical C-HR regions make it difficult to assess the structural transformations of individual gp120 and gp41 subunits. Here, we circumvent this obstacle by studying two types of asymmetric Env trimers. The first type is a heterotrimer of functionally complemented Env protomers, one deficient in CD4 binding and the other deficient in coreceptor binding [34, 35]. The second type is an Env homotrimer containing a gp41 substitution that disrupts three-fold symmetry in the N-HR coiled coil. We explored the exposure of the gp41 ectodomain in these asymmetric Env species using N-HR- and C-HR-targeted fusion inhibitors that block TOH formation. Our results demonstrate that receptor binding does not trigger three-fold symmetric exposure of fusion inhibitor-binding sites as envisioned in the current model. Instead, the findings point to stepwise disengagement of gp120 subunits from gp41 and a gradual uncovering of the trimeric core.

## Results

### Properties of fusion inhibition

Fusion inhibitors bind the gp41 N-HR and C-HR regions exposed in a kinetic window between receptor activation of Env and formation of the gp41 TOH (Fig 1A and 1C) [39–54]. Once bound, they block N-HR/C-HR association and promote irreversible deactivation of the extended gp41 trimer [55]. Due to the transient exposure of these binding sites, fusion inhibitor potency reflects both equilibrium and kinetic factors that describe inhibitor association/dissociation and Env conformational transitions [50, 55]. For a fusion inhibitor that readily dissociates and re-associates during the intermediate-state lifetime, potency is indeed proportional to binding affinity. However, for extremely tight binding fusion inhibitors that rarely dissociate before gp41 deactivates, kinetic factors are the predominant determinant of inhibitory activity [50, 55]. The potencies of such kinetically restricted inhibitors depend on the rate of inhibitor association and lifetime of the sensitive state, but not strictly on affinity. Finally, a kinetically restricted inhibitor can be converted to an affinity-dependent inhibitor in the setting of an extremely destabilizing escape mutation. S1 Table provides an overview of these properties for the fusion inhibitors and Env variants used in this study.

The kinetic properties of fusion inhibition make these inhibitors particularly useful in monitoring late conformational changes in gp41, the duration of the PHI, and the steric environment around the N-HR and C-HR regions during membrane fusion [21, 49, 50, 52, 53, 55–60]. Fusion inhibitors are less useful for resolving early Env structural transitions that lead to N-HR and C-HR exposure. Typically, Env homotrimers present three identical binding sites for a given fusion inhibitor, and deciphering whether these sites are exposed simultaneously or sequentially in the PHI is nearly impossible from inhibitory titrations. We reasoned that this structural information might be gleaned from asymmetric Env trimers in which the three fusion-inhibitor binding sites possessed unequal affinities.

### Engineering asymmetric Env heterotrimers through functional complementation

**Fig 2:**
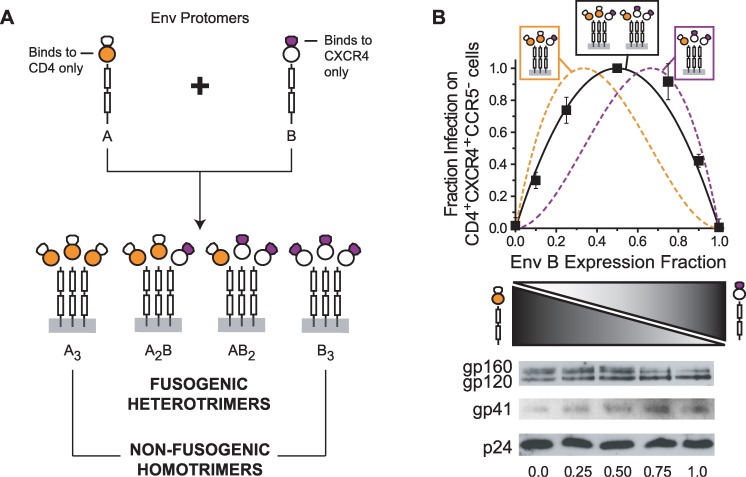
Functional complementation strategy using Env_HXB2_ protomers deficient in either CD4- or CXCR4-binding. (**A**) Env A binds CD4 but cannot interact with CXCR4 due to alterations of its V3 loop. Env B cannot bind CD4 due to a D368R mutation in gp120 but still retains an intact CXCR4-binding site. Co-expression of Envs A and B in HIV-1-producing cells leads to the incorporation of Env homotrimers (non-fusogenic A_3_ and B_3_) and heterotrimers (A_2_B and AB_2_) into budding virus. (**B**) Infectivity of HIV-1 samples produced from cells expressing different ratios of Env A and Env B when total Env levels were held constant. Infection was measured using CD4^+^CXCR4^+^CCR5^-^ target cells and normalized to the p24 content of input virus. The data represent the mean ± SEM of 5 independent experiments, each performed in duplicate and scaled to the maximal measured infectivity (Env B fraction of 0.5). The solid and dotted lines represent the theoretical individual and combined relative populations of the heterotrimeric species in viral samples: orange—A_2_B, purple—AB_2_, black—A_2_B+AB_2_. Theoretical curves were calculated based on a simple binomial model that assumes random protomer assortment and independent incorporation into virus. (**Bottom**) Representative Western blots of Env and p24 on HIV-1 samples used in the infectivity experiments. Virus was prepared from cells transfected with an Env-deficient HIV-1 genome and DNA plasmids encoding Env A and Env B. The total amount of Env-expressing DNA used for transfection was held constant. Viral samples were purified through a sucrose cushion and normalized by p24 content prior to SDS-PAGE. The numbers at the bottom indicate the relative expression of Env B.

As a first step in generating asymmetry in the gp41 trimer, we utilized a functional complementation strategy to produce heterotrimeric Env species. We generated HIV-1 from cells expressing two Env (HXB2 strain) variants, denoted in this paper A and B. Env A was competent in binding CD4 but deficient in binding CXCR4, while Env B was deficient in binding CD4 but competent in binding CXCR4 (Fig 2A, top). In Env A, interaction with CXCR4 was disrupted by swapping the V3 loop from the HXB2 strain with that from the SF162 strain (CCR5-tropic). In Env B, interaction with CD4 was ablated by substituting Asp368 with Arg in the CD4-binding site of gp120 [61, 62]. These alterations did not impact Env expression, processing or incorporation into viruses (Fig 2B). Consistent with previous studies [34, 35], HIV-1 expressing only A_3_ or B_3_ homotrimers were poorly infectious on CD4^+^CXCR4^+^CCR5^-^ target cells (Fig 2B, Env B expression fractions of 0 and 1, respectively). However, HIV-1 generated from cells coexpressing Envs A and B showed substantially higher infectivity, indicating that the functional disruptions in individual protomers could be complemented to generate fusogenic Env heterotrimers A_2_B and AB_2_.

To characterize the fusogenic activities of these Env heterotrimers, we adjusted the relative protomer expression while keeping total Env level in virus producing cells constant (Fig 2B). HIV-1 infectivity levels peaked at a relative Env B expression fraction of 0.5 (an A:B expression ratio of 1:1) and were roughly symmetric around this value. The variance in infectivity closely matched the predicted combined population of both heterotrimers on viruses (Fig 2B, black line), suggesting that these two Env species promoted viral entry with comparable efficiencies. The results implied that Env_HXB2_ trimers with 2 CD4/1 CXCR4 binding sites (A_2_B) and Env_HXB2_ trimers with 1 CD4/2 CXCR4 binding sites (AB_2_) have similar fusogenic activities.

**Fig 3:**
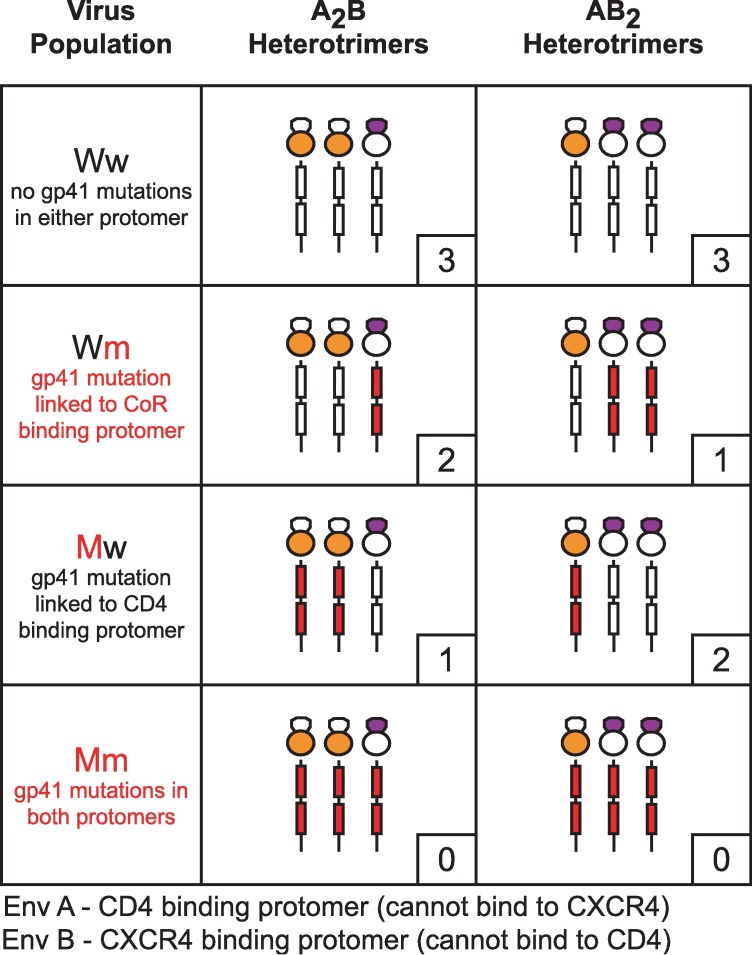
Combining functional complementation with fusion inhibitor escape mutations to assess gp41 exposure. HIV-1 was collected from cells co-expressing Env A and Env B protomers containing either wild type or mutant gp41 subunits. Viral populations are labeled with a two-letter nomenclature as follows: the first letter (upper case) indicates the gp41 status (wild type—W, mutant—M) of the CD4-interacting Env A protomer; the second letter (lower case) indicates the gp41 status (wild type—w, mutant—m) of the coreceptor-binding Env B protomer. The fusogenic A_2_B and AB_2_ heterotrimers for each viral population are schematically depicted with mutant gp41 subunits colored red. The number of high affinity fusion inhibitor binding sites per trimer is indicated in the small box at the lower right. Nonfusogenic Env homotrimers resulting from coexpression of Env A and Env B are not portrayed.

The functional complementation strategy provided a platform to interrogate CD4- or CXCR4-triggered conformational transitions of individual protomers in the context of functional Env trimers. We applied the technique in the next two sections in order to study the transition into the PHI. To assess exposure of the gp41 ectodomain, we introduced escape mutations into the N-HR or C-HR regions and monitored the impact on fusion inhibitor potency (Fig 3). When the coexpressed Envs A and B both had the same gp41 mutation, the viral population (designated Mm) contained functional Env heterotrimers with no high affinity fusion inhibitor binding sites. Similarly, when neither Env protomer had a gp41 mutation, the viral population (designated Ww) contained Env heterotrimers with three high-affinity fusion inhibitor binding sites. A_2_B and AB_2_ heterotrimers on Mm and Ww viruses mimicked mutant and wild type Env homotrimers in terms of symmetry in their gp41 ectodomains. However, when gp41 mutations were incorporated into either Env A or Env B, the viral populations (designated Mw or Wm, respectively) contained functional Env heterotrimers with asymmetric gp41 trimers (Fig 3). These heterotrimers contained either one high affinity / two low affinity binding sites or two high affinity / one low affinity binding site. Importantly for these functional Env trimers, escape mutations were introduced into protomers restricted to either CD4- or CXCR4-binding. In Mw viruses, the escape mutation was introduced into Env A, the protomer that binds CD4. In Wm viruses, the escape mutation was introduced into Env B, the protomer that binds CXCR4. We reasoned that this linkage might allow us to ascertain whether specific fusion-inhibitor binding sites were exposed upon CD4 or CXCR4 binding.

### Inhibitory sensitivities of Env heterotrimers with asymmetric N-HR domains

**Fig 4:**
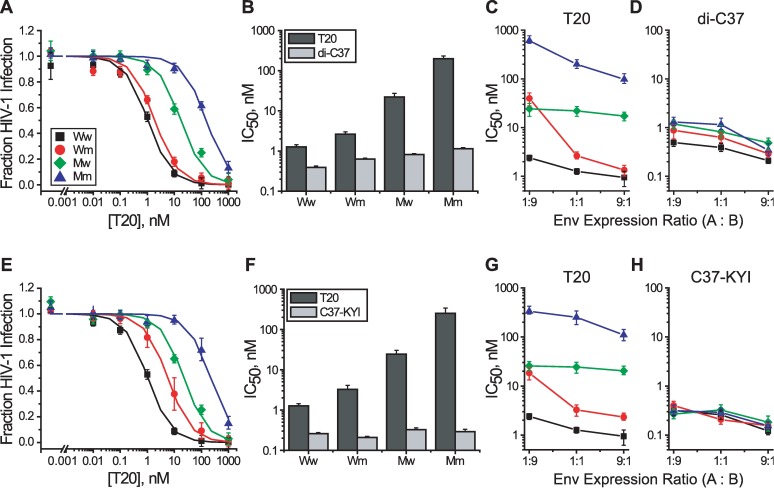
Probing exposure of the gp41 N-HR coiled coil. (**A**) T20 sensitivity of Ww (black), Wm (red), Mw (green) and Mm (blue) viruses where mutant Env protomers contained the L544S substitution. Viral samples were produced from cells expressing equal levels of Env A and Env B protomers. (**B**) IC_50_ values for T20 (dark gray) and di-C37 (light gray) against HIV-1 produced from cells expressing equal levels of Env A and Env B protomers. T20 is affinity-dependent while di-C37 is kinetically restricted. (**C** and **D**) IC_50_ values for T20 (**C**) and di-C37 (**D**) against HIV-1 produced from cells expressing Env A and Env B protomers at the indicated ratios. IC_50_ values (**B**, **C** and **D**) were obtained from fits of inhibitor titration data to the Langmuir equation (solid lines in **A**). (**E-H**) As described for panels **A-D** except that the mutant protomers contained the V549A substitution and C37-KYI was the kinetically restricted inhibitor. Data points represent the means ± SEM from three or more independent experiments. The target cells were U87.CD4.CXCR4.

We probed exposure of the N-HR coiled coil using peptide fusion inhibitors T20, C37-KYI and di-C37 (Fig 1C) [43, 55]. With sequences derived from the gp41 C-HR region, these so called C-peptides bind the hydrophobic groove formed at the interface of two N-HR helices in their coiled-coil conformation [12, 30, 31]. T20 interacts relatively weakly with gp41 and is sensitive to affinity disrupting escape mutations in the N-HR region [63]. C37-KYI and di-C37 bind extremely tightly to gp41 and have kinetically restricted inhibitory potencies that are not sensitive to small affinity disruptions [55]. We employed resistance mutations L544S and V549A that reside within the T20 binding site and decrease C-peptide interaction affinity 50–100 fold (Fig 1C) [50]. Neither point mutation altered Env expression, processing or viral incorporation (S1A Fig and S2A Fig). The V549A mutation had no impact on viral infectivity (S2A Fig), while the L544S mutation had a modest impact on homotrimer infectivity that was not recapitulated in the heterotrimer setting (S1A Fig). In Env_HXB2_ homotrimers, the L544S and V549A substitutions conferred 30- and 50-fold resistance to T20, respectively, but had no impact on the potencies of C37-KYI and di-C37 (S1B and S2B Figs). These inhibitory characteristics were qualitatively preserved in A_2_B and AB_2_ heterotrimers on Ww and Mm viruses, which contain either three high affinity or three low affinity-fusion inhibitor binding sites like wild type and mutant Env homotrimers (Fig 4).

We first tested the inhibitor sensitivities of Env heterotrimers using viral samples generated from cells expressing equal levels of Env A and Env B. Under these conditions, the A_2_B and AB_2_ trimeric species were equally represented and contributed comparably to viral entry. Thus, for the Wm and Mw viral populations, the average fusogenic trimer contained approximately 1.5 high affinity and 1.5 low affinity C-peptide binding sites (Fig 3). We reasoned that if receptor-induced structural changes led to symmetric exposure of the N-HR coiled coil, then Wm and Mw viruses would be similarly sensitive to T20. Instead, we observed that the Wm viruses were 10-fold more sensitive to T20 than Mw viruses (Fig 4). This difference in T20 sensitivity was not due to alterations in Env fusion kinetics as Wm and Mw viruses were equally sensitive to kinetically restricted inhibitors (Fig 4B and 4F and S1 and S2 Figs). Rather, the results suggested that high affinity-binding sites of the N-HR coiled coil were more exposed in Env heterotrimers on Wm viruses than in Env heterotrimers on Mw viruses.

We next asked how the two Env heterotrimeric species, A_2_B and AB_2_, individually contributed to the difference in T20 sensitivity between Wm and Mw viruses. HIV-1 was generated from cells in which the relative expression of Env A to Env B was biased either 1:9 or 9:1. At the 1:9 expression ratio, where the AB_2_ heterotrimer accounted for approximately 90% of the fusogenic Env species, Wm and Mw viruses showed nearly identical sensitivity to T20 (Fig 4C and 4G and S1 and S2 Figs). By contrast, for the 9:1 expression ratio where the A_2_B heterotrimer predominated, Wm viruses were 20-fold more sensitive to T20 than Mw viruses. Thus, the data indicated that the difference in T20 sensitivities of Mw and Wm viruses was primarily due to the A_2_B heterotrimeric species (summarized in Table 1). Again, these differences in T20 sensitivity could not be attributed to alterations in Env fusion kinetics since all viral populations (Ww, Wm, Mw and Mm) were similarly inhibited by kinetically restricted fusion inhibitors (Fig 4D and 4H). Rather, A_2_B heterotrimers on Wm viruses appear to preferentially expose a high affinity site and are potently inhibited by T20 (nearly as well Ww viruses), while the same trimers on Mw viruses appear to preferentially expose a low affinity site and have significantly poorer T20 sensitivity. Hence, A_2_B trimers (2 CD4 / 1 CXCR4 binding site) appear to uncover N-HR regions from CD4-binding Env A protomers before the entire N-HR coiled coil is uncovered. Curiously, AB_2_ trimers (1 CD4 / 2 CXCR4 binding sites) from Wm and Mw viruses had similar T20 sensitivities, as expected if all three T20 binding sites were equally exposed. The results suggested that single CD4 binding to Env leads to symmetric gp41 N-HR exposure (see Discussion).

**Table 1:** Table of Heterotrimers

			gp41 sequence		Inhibitor Sensitivity^#^	
Virus	Inhibitor	Target	Protomer A*	Protomer B^&^	A_2_B Trimer	AB_2_ Trimer
Ww	T20	N-HR	WT	WT	++	++
Wm	T20	N-HR	WT	L544S or V549A	++	+/-
Mw	T20	N-HR	L544S or V549A	WT	+/-	+/-
Mm	T20	N-HR	L544S or V549A	L544S or V549A	-	-
Ww	5H_LAVA_	C-HR	WT	WT	++	++
Wm	5H_LAVA_	C-HR	WT	N656D	++	+
Mw	5H_LAVA_	C-HR	N656D	WT	+	++
Mm	5H_LAVA_	C-HR	N656D	N656D	-	-

*CD4 binding protomer (cannot bind CXCR4)

^&^CXCR4-binding protomer (cannot bind CD4)

^#^IC50 values relative to Ww virus: “++” = within 3-fold; “+” = between 3- and 6-fold; “+/-”= between 8- and 24-fold; “-”= greater than 30-fold

### Inhibitory sensitivities of Env heterotrimers with asymmetric C-HR domains

**Fig 5:**
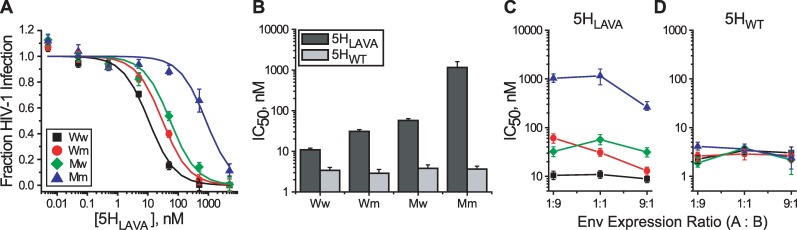
Probing exposure of the three gp41 C-HR domains. (**A**) 5H_LAVA_ sensitivity of Ww (black), Wm (red), Mw (green) and Mm (blue) viruses where mutant Env protomers contained the N656D substitution. Viral samples were produced from cells expressing equal levels of Env A and Env B protomers. (**B**) IC_50_ values for 5H_LAVA_ (dark gray) and 5H_WT_ (light gray) against HIV-1 produced from cells expressing equal levels of Env A and Env B protomers. 5H_LAVA_ is affinity-dependent while 5H_WT_ is kinetically restricted. (**C** and **D**) IC_50_ values for 5H_LAVA_ (**C**) and 5H_WT_ (**D**) against HIV-1 produced from cells expressing Env A and Env B protomers at the indicated ratios. IC_50_ values (**B**, **C** and **D**) were obtained as described in Fig 4. Data points represent the means ± SEM from five independent experiments. The target cells were U87.CD4.CXCR4.

Using the same functional complementation strategy, we examined exposure of the gp41 C-HR using the protein fusion inhibitor 5-Helix (Fig 1C) [44, 50]. The wild type version (denoted 5H_WT_) binds the C-HR region with extremely high affinity and displays kinetically restricted inhibitory potency. A mutant version (denoted 5H_LAVA_) binds less tightly and displays affinity-dependent inhibitory potency. We employed resistance mutation N656D in the C-HR region that reduces 5-Helix binding affinity by more than three orders of magnitude (Fig 1C). Incorporation of the N656D escape mutation did not alter viral infectivity, Env expression or processing (S3A Fig). In Env_HXB2_ homotrimers, N656D conferred more than 100-fold resistance to 5H_LAVA_ but had no impact on the potency of 5H_WT_ (S3B Fig). The same characteristics were observed for Ww and Mm viruses containing A_2_B and AB_2_ heterotrimers (Fig 5).

Unlike our interrogation of T20 inhibition with N-HR escape mutations, no large difference in 5H_LAVA_ inhibition was observed between Wm and Mw viruses (Fig 5, Table 1). The IC_50_ values were within 2-fold of one another at all Env protomer expression ratios. We did observe a small but persistent trend in Wm viruses toward higher sensitivity as the relative fraction of the Env A protomer increased (Fig 5C). That trend was absent in Mw viruses. All viral populations showed similar sensitivity to 5H_WT_ (Fig 5D), indicating that there were no alterations in fusion rates to confound the interpretation of 5H_LAVA_ sensitivities. The data suggested that the high affinity 5-Helix binding sites in heterotrimers on Wm and Mw viruses were similarly exposed, regardless of the stoichiometry of CD4- and CXCR4-binding sites on the Env trimer. Thus, the three gp41 C-HR regions appeared to be uncovered nearly simultaneously, or, at a minimum, much more symmetrically than the T20 binding sites on the N-HR coiled coil.

### Structure of an asymmetric trimer-of-hairpins

The previous experiments took advantage of engineered asymmetry in A_2_B and AB_2_ Env heterotrimers to unmask properties of the native-to-PHI transition. However, it was formally possible that the combination of Env A and B protomers unnaturally produced asymmetric exposure of the gp41 trimer, and that Env homotrimers with fully intact CD4 and coreceptor binding sites would undergo a more symmetric conformational transition. Addressing this concern required an Env homotrimer with a naturally asymmetric gp41 containing high and low affinity-fusion inhibitor binding sites. Towards this goal, we were assisted by the serendipitous discovery that the Q552R substitution introduced asymmetry into the N-HR coiled coil.

**Fig 6:**
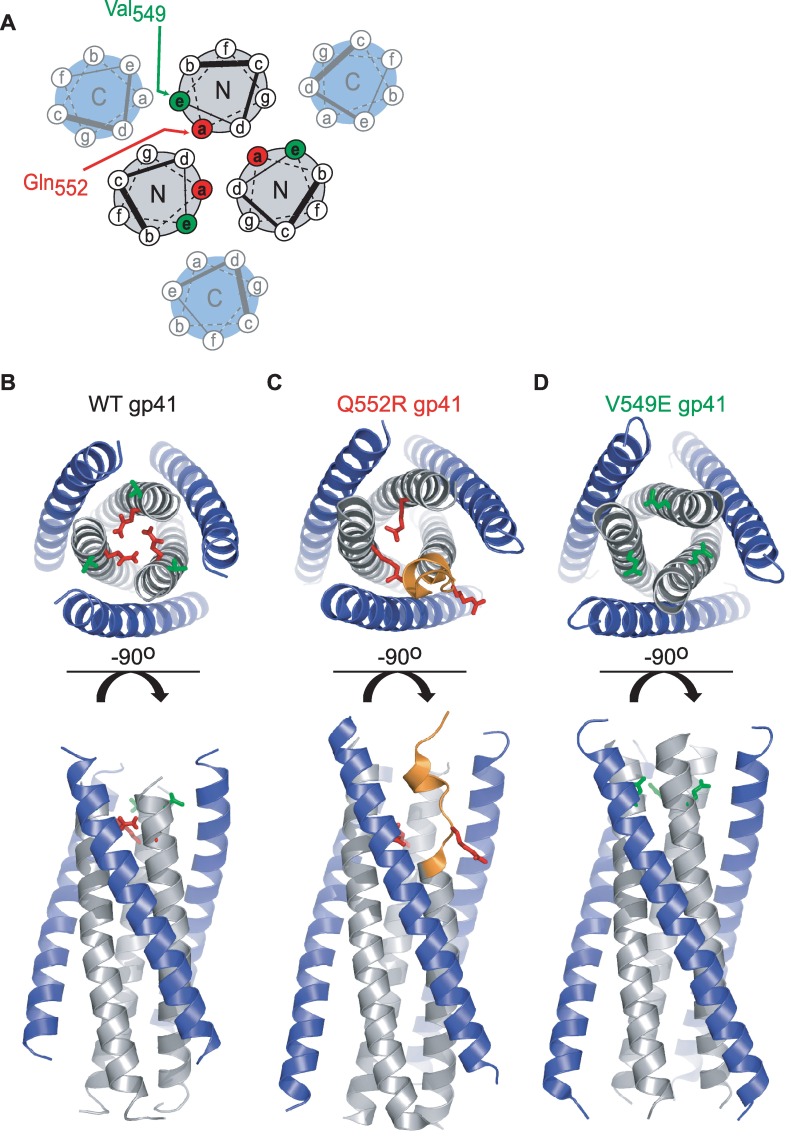
Structures of the wild type and mutant gp41 trimers-of-hairpins. (**A**) Helical wheel diagram of the gp41 trimer-of-hairpins with residue positions labeled *a* through *g* based on the 3,4-hydrophobic heptad repeat. The relative positions of Val_549_ (green) and Gln_552_ (red) within the N-HR coiled coil are designated. (**B**) Ribbon diagram of the wild type gp41 trimer-of-hairpins formed by peptides N36 (gray) and C34 (blue) based on the Env_HXB2_ sequence (PDB ID: 1AIK. [12]). The side chains of Val_549_ (green) and Gln_552_ (red) are depicted in stick representation. (**C**) Ribbon diagram of a gp41 trimer-of-hairpins containing the Q552R substitution. The side chains of Arg_552_ are shown in stick representation (red) while the unwound region of the distorted N40 segment is colored orange. (**D**) Ribbon diagram of the gp41 trimer-of-hairpins containing the V549E substitution. The side chains of Glu_549_ are shown in stick representation (green). The structures in **C** and **D** were obtained using a gp41 construct (NC1) containing 40 amino acids of the N-HR segment (gray) and 37 amino acids of the C-HR segment (blue).

The thermostable core of the gp41 TOH consists of the three-stranded N-HR coiled coil surrounded by three helical C-HR segments (Fig 6A) [12, 30, 31]. Each C-HR segment packs into a hydrophobic groove formed at the interface of two different N-HR helices. The wild type structure displays an overall three-fold symmetry parallel to the longitudinal axis of the coiled coil (Fig 6B). Notably, the Gln_552_ residues of each N-HR helix (in the *a*-position of the hydrophobic heptad repeat, see Fig 6A) point toward the hydrophobic interior of the coiled coil, with their side chain amides forming hydrogen bonds with backbone carbonyls on neighboring helices (Fig 6B).

Propagating HIV-1 (NL4-3 strain) in the presence of fusion inhibitor C37-KYI led to the selection of the escape mutation Q552R. With its additional bulk and positive charge, we suspected that the Arg_552_ side chains would not adopt the same configuration in the gp41 TOH as observed for Gln_552_. To determine the structural impact of this mutation, we crystallized a construct composed of 40 residues from the N-HR sequence (N40) and 37 residues from the C-HR sequence (C37) and solved its structure (Fig 6C, S2 Table). Although the mutant six-helix bundle crystallized in a hexagonal space group (P3_1_), three-fold crystallographic symmetry did not run through the center of the N-HR coiled coil as it does in almost all previous gp41 TOH structures. Instead, the structure was markedly asymmetric in that only two of the three N40 segments were folded into α-helices throughout their entire length. The N-terminal region (residues 545–554) of the third N40 segment assumed a distorted, largely extended conformation (Fig 6C, S4A Fig). However, by residue 555 (three residues C-terminal to the Q552R mutation), this N40 segment resumed a helical fold, restoring three-fold symmetry to the coiled coil. This symmetry was maintained throughout the remainder of the molecule (S4C and S4D Fig).

As a consequence of the asymmetric distortion of the N-HR coiled coil, the three Arg_552_ residues occupied distinctly different chemical environments. One Arg_552_ side chain extended from an intact N-HR helix into the coiled-coil interior, where it formed a hydrogen bond with the backbone of the other intact N-HR helix (S4B and S5 Figs). The Arg_552_ side chain from the second intact N-HR helix reached into an N40/C37 interface, replacing the Gln_551_ side chain from an adjacent N-HR segment. The third Arg_552_ side chain extended from the distorted N-HR segment (next to the displaced Gln_551_) radially away from the center of the TOH. We speculated that the spatial arrangement of the three Arg_552_ side chains minimized their electrostatic repulsion, which would be particularly destabilizing if all three were confined within the interior of the N-HR coiled coil.

Unlike the N40 coiled coil, all three C37 peptides in the Q552R-mutant TOH structure folded into their normal helical structures, even in the asymmetric zone of the N40 segment (Fig 6C). The backbone conformation of these C-HR segments closely mimicked that for the wild type six-helix bundle (RMSD = 0.34 Å, S6 Fig). The lack of distortion in the C37 main chains was surprising, but might have resulted from constraints imposed by crystal contacts with neighboring six-helix bundles. Despite their similar structures, the three C37 helices were unlikely to have equal affinities for the Q552R-mutant coiled coil. One C37 helix bound a groove formed by two intact N40 helices and was stabilized by the same N-HR/C-HR contacts observed in the wild type TOH (S5 Fig). By contrast, the other two C37 helices bound into grooves formed by one intact N40 helix and the distorted N40 helix, resulting in the loss of a number of stabilizing interactions (S5 Fig). Thus, in contrast to the wild type N-HR coiled coil, the Q552R-mutant coiled coil possessed dissimilar C37 binding sites, with the distorted N-HR segment substantially disrupting two out of the three sites.

To determine if asymmetry in TOH structure is a definitive feature of HIV-1 resistance to C37, we also crystallized an N40/C37 construct containing the V549E substitution (Fig 6D, S1 Table). Residue 549 (in an *e*-position of the N-HR hydrophobic heptad repeat, see Fig 6A) extends directly into the N-HR/C-HR interface where it fills a small hydrophobic pocket bounded by side chains of Asn_656_, Glu_657_ and Leu_660_ and the backbone of the C-HR helix (S7A and S7B Fig). The six-helix bundle containing the V594E mutation crystallized in a rhombohedral space group (R3) with a defined three-fold crystallographic symmetry down the axis of the N40 coiled coil. The V549E substitution did not disrupt the continuity of the N40 coiled coil nor C37 docking on its surface (Fig 6D, S7C and S7D Fig). Overall, the mutant structure closely resembled the wild type six-helix bundle (RMSD = 0.94 Å, S6 Fig). Symmetry dictated that the three C37 peptides bind the V549E-mutant coiled coil with equal affinity, which will be lower than that for the wild type coiled coil due to disrupted hydrophobic packing. Hence, asymmetry is not a general feature of escape from C37-like fusion inhibitors, but rather a special property of the Q552R substitution.

### Inhibitory sensitivities of Q552R and V549E Env variants

As with the previous functional complementation experiments, we used fusion inhibitors to probe gp41 exposure in wild type and mutant Env homotrimers. We reasoned that the presence of both high and low affinity binding sites on the Q552R N-HR coiled coil would manifest in the shape of the inhibitor titration if the sites were sequentially uncovered, but not if the entire coiled coil was symmetrically exposed. HIV-1 was pseudotyped with wild type Env or one of two resistant variants, Env_Q552R/N637K_ and Env_V549E/N637K_. The additional N637K substitution in the C-HR region stabilized the N-HR/C-HR interaction and supported the fusogenic activity of Envs containing affinity-disrupting N-HR mutations (especially the Q552R substitution) [64, 65]. For these experiments, we employed fusion inhibitors C37-KYI, di-C37, 5H_WT_ and PIE12 (Fig 1C, S1 Table). Inhibition by C37-KYI is kinetically restricted against Env_WT_, but affinity-dependent against the two resistant Envs [55]. Inhibition by di-C37 and 5H_WT_ is kinetically restricted against all three Envs [55]. PIE12 is an affinity dependent D-peptide inhibitor that targets the C-terminal region of the N-HR coiled coil [66]. The inhibitor combination allowed us to monitor both binding site exposure and Env conformational dynamics around the site of escape mutations and in regions distal to them.

**Fig 7:**
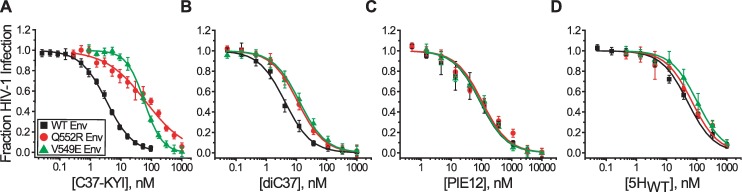
Effect of escape mutations Q552R and V549E on fusion inhibitor activity. HIV-1 was pseudotyped with wild type Env_HXB2_ (black) or Env variants containing the Q552R/N637K (red) or V549E/N637K (green) substitutions. Inhibitor titrations were performed with C37-KYI (**A**), di-C37 (**B**), PIE12 (**C**), and 5H_WT_ (**D**) using HOS-CXCR4+ target cells. The regions of gp41 targeted by these inhibitors are shown in Fig 1C. Data points represent the mean ± SEM of three to seven independent experiments. In **A**, solid lines for wild type Env and the V549E mutant variant reflect fits of the data to a Hill equation; the red line for the Q552R mutant Env is a fit of the data to an inhibition model with both high and low affinity C37-KYI binding sites (see text). In **B**, **C** and **D**, the data have been fit to a simple Langmuir equation.

The most striking feature of these inhibition experiments was the nonparallel dose-response curves for the three C37-KYI titrations (Fig 7A). The titration for Env_V549E/N637K_ was steeper than that for Env_WT_, while the titration for Env_Q552R/N637K_ was substantially less steep. This variance in slope caused the inhibition curves of the two resistant Envs to intersect near their IC_50_ values. This observation is unique to the C37-KYI titrations, as inhibition by di-C37, 5-Helix and PIE12 yielded parallel dose-response curves for all three Env species (Fig 7B–7D). These results implied that the slope difference observed for C37-KYI was not due to alterations in Env transition kinetics, nor was it a general property of affinity-dependent fusion inhibitors targeting these three Env species. Rather, the effect was specific for an affinity-dependent inhibitor that binds the N-terminal region of the N-HR coiled coil.

To quantify the slope difference, we initially fit C37-KYI inhibition data to the Hill equation and extracted Hill coefficients n_H_. The titration curve for Env_WT_ yielded an n_H_ of 1 (Fig 7A, solid black line), implying a single C37-KYI blocked viral entry. The data were consistent with one C37 peptide being sufficient to disrupt the function of an Env trimer (KH and MJR, submitted). Since C37-KYI is kinetically restricted against Env_WT_ and, therefore, does not dissociate before gp41 deactivates, the binding of a second or third C37-KYI would not alter inhibition probability. Indeed, inhibition data for kinetically restricted fusion inhibitors di-C37 and 5-Helix similarly fit with an n_H_ of 1 (Fig 7B and 7D, all lines). The C37-KYI titration curve for Env_V549E/N637K_ yielded an n_H_ of 1.45 (Fig 7A, solid green line), indicating that a significant fraction of inhibition events involved more than one bound peptide. This observation likely reflected the reduced binding affinity for C37-KYI causing the inhibitor to dissociate more rapidly than Env_V549E/N637K_ could deactivate; in this situation, inhibition probability would be enhanced with more than one peptide bound per trimer. The titration curve for Env_Q552R/N637K_ yielded an n_H_ of 0.67, substantially less than the Hill coefficients from both the kinetically restricted inhibition of Env_WT_ and affinity-dependent inhibition of Env_V549E/N637K_. The low n_H_ value suggested that C37-KYI inhibited Env_Q552R/N637K_ by interacting with binding sites of different affinity. Therefore, we reanalyzed the data assuming that C37-KYI inhibited a portion of Env_Q552R/N637K_ trimers with high potency and the remainder with low potency. In this case, infection probability (P_I_) can be quantified in terms of two IC_50_ values (IC_50.1_ and IC_50.2_) and the C37-KYI concentration ([Inh]):

$$P_{I}=f\left(\frac{IC_{50.1}}{[Inh]+IC_{50.1}}\right)+\left(1-f\right)\left(\frac{IC_{50.2}}{[Inh]+IC_{50.2}}\right)$$ (1)

where *f* represents the fraction of inhibition events that titrate with IC_50.1_. The data fit best with *f* = 0.31, IC_50.1_ = 8 nM, and IC_50.2_ = 180 nM (Fig 7A, solid red line). The results suggested that one third of inhibition events involved binding to a high affinity site, while two thirds involved binding to low affinity sites.

The multiphasic titration curve for Env_Q552R/N637K_ suggested that its N-HR coiled coil adopts the asymmetric conformation observed in the crystal structure of the mutant TOH. More significantly, the data also pointed to sequential, stochastic exposure of C37-binding sites during membrane fusion. If the N-HR coiled coil were exposed symmetrically following a concerted structural change of the Env trimer, then C37-KYI would always be able to bind its high affinity site, and the titration would be monophasic as observed for Env_WT_ and Env_V549E/N637K_. Rather, the data suggested that this high affinity site was only fully exposed for one third of Envs involved in viral membrane fusion. The remainder of the Envs fully exposed a low affinity site. Such a result is expected if receptor-induced structural changes in Env randomly uncovered a single C37-binding site, with subsequent structural changes required to expose the remainder of the N-HR coiled coil in preparation for gp41 collapse into the TOH.

## Discussion

CD4 and chemokine receptor (CoR) binding guide the structural transformation of HIV-1 Env from a constrained, metastable native state through an extended PHI configuration and ultimately into a compact, low energy TOH conformation [15, 28]. Structures of the native Env trimer reveal how the gp120 canopy effectively restrains gp41 subunits from premature triggering, while structures of the TOH divulge its role in juxtaposing the viral and cellular membranes for fusion [6, 7, 12, 30, 31]. Unfortunately, the structure of Env in the PHI and the disposition of gp120 subunits during this state remain unknown. Evidence from temperature and lipid arrest states of Env-mediated membrane fusion and from fusion inhibitor synergy measurements suggest the PHI might itself be a combination of multiple evolving states, with the N-HR region becoming progressively exposed and the C-HR region becoming progressively occluded as fusion proceeds [52, 55]. Our experiments here asked whether the gp41 trimer was revealed in a three-fold symmetric manner upon entry into the PHI, or whether the N-HR coiled coil and three C-HR regions were uncovered in piecemeal fashion.

To interrogate these early conformational transitions, we employed functionally complemented Env heterotrimers consisting of protomers deficient in either CD4- or CXCR4-binding. Fusion inhibitor-escape mutations were then selectively introduced into either protomer, and the impact on inhibitor potency was measured. For N-HR-targeted T20, potency depended on whether the escape mutation was incorporated into the CD4- or CXCR4-binding protomer. By contrast, for C-HR-targeted 5H_LAVA_, potency was largely unaffected by which protomer contained the mutation. The results suggested that the C-peptide binding sites on the gp41 N-HR coiled coil were exposed in piecemeal fashion, while the gp41 C-HR regions were exposed symmetrically. Our interpretations were based on experiments complicated by multiple levels of Env manipulation (e.g., functional complementation, fusion inhibitor resistance and biased protomer expression). Such alterations, and perhaps even fusion inhibitor binding itself, could have shifted the conformational landscape of the trimer and played a more active role in promoting what appeared to be an asymmetric transition. It was also possible that our observations were a consequence of the fact that CD4 and CoR could not bind the same protomer in the Env heterotrimer. To partially address these concerns, we analyzed the fusion inhibitor sensitivity of a naturally asymmetric Env homotrimer (Env_Q552R/N637K_) that has three intact CD4 and CXCR4 binding sites. Inhibitor titrations revealed that C37-KYI blocked viral entry with multiple potencies, as if the C-peptide bound to sites of different affinities and these sites were asymmetrically exposed. These measurements of viral entry inhibition remain an indirect reporter of Env structural changes. More direct methods, such as cryo-EM structural determination combined with inhibitor binding/precipitation assays, will be required to confirm our results.

Our data suggest that entry into the PHI leads to near simultaneous exposure of the three gp41 C-HR regions but only uncovers a portion of the gp41 N-HR coiled coil. The most likely structure occluding C-peptide binding sites is gp120, but how this protein remains bound to gp41 in the PHI is unclear. In native Env, the interior of each gp120 subunit cradles a gp41 N-terminal region that adopts a conformation vastly different from the one envisioned for the PHI state (Fig 1B) [6, 8–11]. The N-HR domain is segmented into two helical regions (residues 548–568, 571–593), an extended loop preceding the first helix (534–547) and a short linker connecting the two helices (569–570) [10]. The two helical segments run roughly antiparallel to one another, with the C-terminal helix from each N-HR domain assuming a coiled-coil conformation. While the C-terminal helix partially interfaces with gp120, the N-terminal helix and extended loop make extensive contacts with the surface subunit (especially its C1 domain). For the entire N-HR region to adopt the coiled-coil structure and form fusion inhibitor binding sites, each gp120 subunit would need to disengage fully from the N-terminal helix, extended loop and gp41 N-terminus (fusion peptide). This structural rearrangement potentially destabilizes the gp120-gp41 interaction. Indeed, addition of soluble CD4 to Env_HXB2_ (the viral strain used in these experiments) is sufficient to promote both N-HR coiled-coil formation and rapid shedding of gp120 [39, 48, 54, 67, 68]. However, if receptor triggered entry into the PHI caused all three gp120 subunits to disengage completely, then the three fusion inhibitor binding sites would be exposed simultaneously. Our results suggest that Env trimers actively involved in viral membrane fusion retain at least some of their gp120 subunits well after the N-HR coiled coil has formed.

The T20 sensitivities of biased heterotrimers (Fig 4C and 4G) suggest that receptor binding stoichiometry plays a role in determining how the gp41 N-HR coiled coil becomes exposed. In AB_2_ heterotrimers (1 CD4 / 2 CXCR4 binding sites), the three C-peptide binding sites on the N-HR coiled coil appear to be exposed equally (reflected by the similar T20 potency against Wm and Mw viruses, Table 1). By contrast, in A_2_B heterotrimers (2 CD4 / 1 CXCR4 binding site), the C-peptide binding sites appear to be uncovered asymmetrically (reflected by the different T20 potency against Wm and Mw viruses, Table 1). Formally, either multiple CD4 binding or single CXCR4 binding or both could be responsible for the asymmetric exposure of the N-HR coiled coil in A_2_B heterotrimers. However, the multiphasic C37-KYI titration of Env_Q552R/N637K_ suggests that trimers with 3 CD4 binding sites and 3 CXCR4 binding sites also reveal their N-HR coiled coil in piecemeal fashion. Although our experiments do not directly report on receptor interaction stoichiometry during viral entry, the findings point to multiple CD4 binding as the primary driver of an asymmetric transition into the PHI.

Although asymmetric exposure of gp41 appears to require at least two CD4 interactions with Env, our data suggest that these binding events uncover only a single C-peptide binding site. This conclusion explains how C37-KYI seems to bind a high affinity site only one-third of the time when inhibiting Env_Q552R/N637K_ homotrimers. If, instead, asymmetric exposure of the N-HR coiled coil revealed two C-peptide binding sites, then the high affinity site would be exposed two-thirds of the time and two-thirds of inhibition events would be of high potency. Likewise, the similar impact of the L544S and V549A substitutions in our functional complementation experiments can be best understood if only a single site is exposed. Residues 544 and 549 point into different C-peptide binding grooves on the N-HR coiled coil (S8 Fig), and, therefore, mutant heterotrimers display distinct organizations of low and high affinity C-peptide binding sites. As shown in S8 Fig for A_2_B heterotrimers of Mw viruses, simultaneous exposure of two C-peptide binding sites would reveal two low affinity sites for one mutation but would uncover one low affinity and one high affinity site for the other mutation. However, for both L544S and V549A substitutions, A_2_B heterotrimers on Mw viruses are insensitive to T20. Thus, our data are most consistent with exposure of a single site on the N-HR coiled coil of A_2_B heterotrimers, specifically the one common to both L544S and V549A substitutions and formed by the two Env A protomers (S8 Fig).

**Fig 8:**
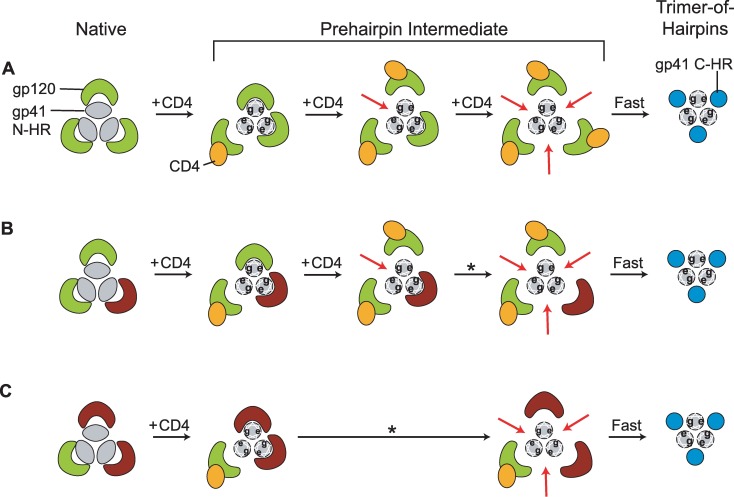
Hypothetical model of the early structural changes in Env leading to exposure of the N-HR coiled coil. (**A**) Proposed transitions for an Env trimer that encounters three CD4 molecules. In the native state, gp120 subunits (green) constrain the N-HR segments (grey) in a metastable conformation in which the coiled coil is only partially formed (schematically represented as ovals). The binding of the first CD4 (orange) alters trimer structure enabling formation of the full N-HR coiled coil (schematically represented as circles with *e*- and *g*-positions of the heptad repeat designated). The CD4-bound gp120 subunit disengages but only partially exposes C-peptide binding sites on either side of its cognate N-HR helix. The remaining two gp120 subunits remain closely associated with the coiled coil, thereby blocking C-peptide access to all three binding sites. The second CD4-gp120 interaction causes this gp120 subunit to disengage, opening up a single C-peptide binding site on the N-HR coiled coil (designated by the red arrow). The other two C-peptide binding sites remain partially occluded by the one gp120 that remains attached. Upon association of the third CD4, the last gp120 disengages and the N-HR coiled coil with its three C-peptide binding sites becomes fully exposed. Ultimately, these sites become occupied by gp41 C-HR regions (blue) as Env transitions into its fusogenic trimer-of-hairpins conformation. The model describes the putative transitions of homotrimeric Envs, including the Q552R/N637K variant of Fig 7. (**B** and **C**) Proposed transitions for an Env trimer that encounters only two (**B**) or one (**C**) CD4 molecule. The gp120 subunits that do not interact with CD4 are colored brown. The asterisk indicates a spontaneous, CD4-independent conformational change. The models describe the putative transitions of A_2_B (**B**) and AB_2_ (**C**) heterotrimers.

In Fig 8, we present a working model of early Env structural changes that describes how multiple CD4 binding might uncover a single C-peptide binding site on the N-HR coiled coil. We speculate that an unliganded gp120 subunit remains closely associated with its cognate N-HR helix following coiled-coil formation. This association spatially restricts access to the two C-peptide binding sites on either side of the N-HR helix. CD4 binding leads gp120 to detach from the gp41 N-terminal region and sterically unveil the two previously occluded half-sites on the coiled coil. The first CD4 interaction with the Env trimer might trigger N-HR coiled-coil formation, but no C-peptide binding site would be completely exposed since the two unliganded gp120 subunits remain attached. Alternatively, formation of the N-HR coiled coil and, by extension, the three C-peptide binding sites, might not even occur until the second CD4 binds [9]. In either case, interaction of this second CD4 would uncover a single site, the one formed by the N-HR helices of the two CD4-bound protomers. The other two sites would continue to be partially occluded by the remaining unliganded gp120 subunit. Interaction of a third CD4 would lead to a fully exposed N-HR coiled coil, a thermodynamically unstable conformation that likely resolves rapidly into the TOH (Fig 8A). The short lifetime of this fully exposed state limits C-peptide binding to the two previously occluded sites, reducing their impact on inhibitory potency. Thus, the properties of the first exposed site will have the greatest impact on C-peptide inhibitory potency. This model explains how C37-KYI inhibits Env_Q552R/N637K_ homotrimers with varying potencies despite all trimers having the same complement of C-peptide binding sites. Although the model ties receptor binding to g41 exposure, it is important to emphasize that our experiments did not directly measure CD4 interaction with Env and that all gp120 subunits may not engage CD4 prior to viral membrane fusion. The model does not address the role of chemokine receptor binding in coordinating structural changes, nor does it precisely define when the N-HR coiled coil forms during early Env activation. Current research in our lab is attempting to tackle these shortcomings.

Although the model portrays three CD4 interactions, Env trimers can function binding fewer CD4 molecules (and chemokine receptors) (Fig 2) [34–36, 69]. Despite having only one or two CD4 binding sites (and 2 or 1 CXCR4 binding sites, respectively), A_2_B and AB_2_ heterotrimers have robust fusogenic activities compared to Env_HXB2_ homotrimers (S1A and S2A Figs). The proposed model explains how Wm and Mw viruses have such different sensitivities to T20 when A_2_B heterotrimers, with 2 CD4 binding sites, predominate (Fig 8B). Following the second CD4-gp120 interaction, the C-peptide binding site formed by N-HR helices from both Env A protomers is uncovered. When Env A protomers have wild type gp41 subunits, as in Wm viruses, the exposed site has a high affinity for C-peptides and the trimer is sensitive. Conversely, when the Env A protomers contain an escape mutation, as in Mw virus, the exposed site has a low affinity and the trimer is resistant. Full exposure of the remaining two C-peptide binding sites requires spontaneous, CD4-independent detachment of the final gp120 subunit, but their impact on potency is limited by the short lifetime of this state.

The proposed model may also explain how Wm and Mw viruses show similar sensitivities to T20 when AB_2_ heterotrimers, with only 1 CD4 binding site, predominate. Exposure of any C-peptide binding site on these trimers requires spontaneous detachment of at least one Env B gp120 subunit from the N-HR coiled coil (Fig 8C). The similar sensitivities of Wm and Mw viruses imply that wild type and mutant C-peptide binding sites are equally accessible during the PHI regardless of whether the escape mutation is introduced into Env A or Env B. Equal accessibility would be achieved if both Env B gp120 subunits detached nearly simultaneously (Fig 8C). The instability of the resulting fully exposed gp41 trimer would then limit T20 binding, rendering AB_2_ trimers on Wm and Mw viruses similarly resistant. Alternatively, spontaneous detachment may not be as unidirectional as illustrated in the model, and unliganded gp120 subunits might repeatedly detach and reattach during the lifetime of the PHI. In this case, exposure of high and low affinity C-peptide binding sites would be equal from a time-averaged perspective (as opposed to the spatial perspective as proposed above). Additional experiments will be required to distinguish these possibilities.

Interestingly, N-HR regions from other class 1 viral fusion glycoproteins are segmented in their native, prefusion conformation similar to HIV-1 Env. In the hemagglutinin (HA) protein of influenza virus, each N-HR region is broken into two antiparallel alpha-helices, with the C-terminal helix participating in a coiled coil and the N-terminal helix encapsulated by the surface subunit to maintain the metastable conformation (S9A Fig) [70]. In glycoprotein GP from Ebola virus, the N-HR region is multiply segmented, forming a C-terminal helix that participates in a short coiled coil while a number of preceding helical and extended fragments snake around the surface subunit (S9B Fig) [71]. Like gp120, the surface subunits of HA and GP would need to disengage from the N-terminal regions of the transmembrane subunits in order for the N-HR coiled coil to form. Since a disulfide bond crosslinks the surface and transmembrane subunits of HA and GP, disengagement would not lead to surface subunit dissociation and shedding upon entry into the PHI. It remains to be seen if covalent intersubunit association helps to guide these fusion glycoproteins through symmetric conformational transformations, or if the three-fold symmetry of these structures is lost at least for a short time early in the fusion process, as suggested for HIV-1 Env.

In developing an HIV-1 vaccine, the gp41 N-HR coiled coil has always been imagined as a viable target for neutralizing antibodies based on how well C-peptides inhibit viral entry [33]. Toward this goal, vaccination and selection experiments employing engineered forms of the N-HR coiled coil have been successful at generating antibodies that strongly interact with the antigens [72–77]. However, these studies have largely failed to identify an antibody with broad and potent neutralizing activity. Sterically restricted access to the N-HR coiled coil has been implicated as one potential reason for poor neutralization activity [57]. Our results here suggest a more fundamental problem with the antigen itself. The symmetrically exposed N-HR coiled coil that mimics the antigenic structure appears to be available for only a short period during the PHI. During the state primarily targeted by C-peptides, the N-HR coiled coil seems to be asymmetrically exposed, with one binding site uncovered and two binding sites partially occluded. Antibodies recognizing this conformation should have a better chance of binding gp41 during the PHI. Generating such antibodies might require engineering an asymmetric antigen containing an N-HR coiled coil and, perhaps, one or two attached, unliganded gp120 molecules.

## Materials and Methods

### Cell lines and reagents

The following cell lines and reagents were obtained through the NIH AIDS Reagent Program, Division of AIDS, NIAID, NIH: U87.CD4.CXCR4 from Drs. HongKui Deng and Dr. Dan R. Littman [79]; HOS-CXCR4+ from Dr. Nathaniel Landau [78]; pCAGGS_SF162gp160 (expression vector for Env_SF162_) from Drs. L. Stamatatos and C. Cheng-Mayer [80]; Chessie 8 from Dr. George Lewis [81]; HIV-Ig (Catalog number 3957) from NABI and NHLBI. HEK 293T cells and hybridomas for anti-gp120 antibodies 55–83 and 46–2 were obtained from the ATCC. Entry inhibitor PIE12 was a kind gift of Dr. Michael Kay (University of Utah) [66].

### Molecular biology

Variants of the HIV-1_HXB2_ *env* gene were generated in the expression plasmid pEBB_Env_HXB2_ [82]. To make the Env A protomer for functional complementation experiments, a DNA cassette of the V3-loop sequence from Env_SF162_ was produced by PCR (Pfu Polymerase, Stratagene) using pCAGGS_SF162gp160. The cassette was then utilized in a QuikChange reaction (Stratagene) to swap the V3-loop sequence from Env_HXB2_ with that from Env_SF162_. The aligned protein sequences of the two gp120 V3 loops are listed below with amino acid differences underlined:

HXB2 (residues 296–331): CTRPNNNTRKRIRIQRGPGRAFVTIGK-IGNMRQAHC

SF162 (residues 294–327): CTRPNNNTRKSITI--GPGRAFYATGDIIGDIRQAHC

Additional point mutations were made through QuikChange PCR using standard overlapping oligonucleotides (Integrated DNA Technologies):

1. D368R in the CD4-binding site of gp120 to produce the Env B protomer for functional complementation studies,
2. L544S and V549A (T20 resistance mutations) in the gp41 N-HR region of Env_HXB2_, Env A and Env B;
3. N656D (5H_LAVA_ resistance mutation) in the gp41 C-HR region of Env_HXB2_, Env A and Env B; and
4. V549E and Q552R in the gp41 N-HR region and N637K in the gp41 C-HR region (C37-KYI resistance mutations) of Env_HXB2_.

All designed Env constructs were confirmed by DNA sequencing the entire open-reading frame (Kimmel Cancer Center Cancer Genomics Facility, Thomas Jefferson University). Plasmid DNA was prepared for transfection using Promega purification kits and DNA concentrations were quantified by absorbance at 260 nm.

### Analyzing Env expression

To ensure the accuracy of Env expression ratios for functional complementation experiments, we assessed the impact of sequence modifications on the cellular expression of Env A, Env B and Env variants containing T20 or 5H_LAVA_ escape mutations. Env expression was assessed in 293T cells cotransfected (Lipofectamine, Life Sciences) with the Env-deficient HIV-1_NL4-3_ genome (pNL4-3R^-^E^-^Luc^+^) [82] and either one or two Env-expressing plasmids. Virus-containing supernatants were harvested 48 hours post-transfection and characterized as described below. Cells were washed extensively with phosphate buffered saline (PBS, pH 7.4) to remove uncollected virus and cellular debris before being lysed with 1% Triton in PBS supplemented with protease inhibitors (Complete tablets, Roche). Lysates were clarified by centrifugation (10,000 x g, 10 minutes) and the HIV-1 p24 antigen content was determined by ELISA (Aalto, Ireland). Samples of equal p24 protein content were separated by SDS-PAGE, transferred to nitrocellulose paper (Novex, Life Sciences; Hybond C, GE), and probed with 1) monoclonal antibody Chessie 8, which recognizes an epitope in the gp41 cytoplasmic tail; 2) monoclonal antibodies 55–83 and 46–2, which recognize the C1 and C5 constant regions of gp120, respectively; and 3) primary polyclonal HIV-Ig, which detects, among other viral proteins, p24 capsid. Western blots were developed with an HRP-conjugated secondary antibody (Jackson ImmunoResearch) and ECL reagent kit (Pierce). Env A, Env B and Env variants containing L544S, V549A or N656D mutations were found to express at the same levels as wild type Env_HXB2_ (S1, S2 and S3 Figs).

### Characterization of pseudotyped HIV-1 particles

HIV-1 particles were prepared from 293T cells (5x10^6^) transfected with a constant amount of pNL4-3R^-^E^-^Luc^+^ DNA (2.5 μg) and one or two Env expressing plasmids (2.5μg total). For functional complementation experiments, plasmids encoding Env A and Env B constructs were premixed at molar ratios that equaled the indicated expression ratios. Supernatants containing viral particles were harvested 48 hours post-transfection and centrifuged (2500 x g, 10 minutes) to remove cellular debris. Virus was further purified through a 20% sucrose cushion [PBS, pH 7.4, with 2% w/v bovine serum albumin (BSA)] by ultracentrifugation (Beckman L-80, SW 50.1 rotor, 150,000 x g, 70 minutes, 4°C). Pellets were resuspended in PBS with 2% BSA, and viral titers were quantified by p24 antigen ELISA. To assess Env incorporation into virus, samples containing equal p24 content were separated by SDS-PAGE and analyzed by Western Blot as described above. The Env modifications used in this study did not alter the incorporation of Env into the HIV-1 particles (Fig 2B). To assess the fusogenic activities of different Env trimer populations, viral samples were applied to U87.CD4.CXCR4 target cells and infectivity was quantified 48 hours later by expression of a luciferase reporter construct (Promega, Pierce). Luciferase activity was normalized by the p24 content of the input viral sample in order to determine absolute fusogenicity.

### Inhibitor preparation and inhibition studies

C-peptide T20 was synthesized using standard Fmoc chemistry by the peptide synthesis facility at the Kimmel Cancer Center, Thomas Jefferson University. Cleaved, desalted peptides were purified to homogeneity by reverse phase high pressure liquid chromatography (rp-HPLC) using a Vydac C-18 column and a linear gradient of acetonitrile in water containing 0.1% trifluoroacetic acid. The identity of T20 was confirmed using MALDI-TOF mass spectrometry.

C-peptides C37, C37-KYI and di-C37 were prepared from a recombinant gp41 TOH construct NC1 as previously described [44, 55]. NC1 contains a 40 residue N-HR segment (amino acids 543–582, Env_HXB2_ sequence), a trypsin-cleavable linker (GGRGG), a 37-residue C-HR segment (625–661) and a C-terminal hexahistidine tag (GHHHHHH). Briefly, the protein was expressed in *E*. *coli* RP3098 and purified from lysates using metal chelate chromatography (Ni-NTA agarose, Qiagen). The trimeric protein was subject to trypsin digestion (Sigma, 1:250 mass ratio, overnight at 4°C), and C37 was purified to homogeneity using rp-HPLC as described above. C37-KYI contains the affinity enhancing mutations N637K/T639I. Di-C37 is a dimerized form of C37 connected via an engineered disulfide bond at the C-terminus of each monomer.

5-Helix proteins were recombinantly expressed and purified from *E*. *coli* RP3098 as previously described [44, 50]. 5-Helix contains three N40 segments and two C37 segments alternately connected into a single polypeptide. The proteins were solubilized from bacterial inclusion bodies using 8 M guanidine HCl (GdnHCl) in tris-buffered saline (TBS, pH 8), loaded onto Ni-NTA agarose beads, and renatured in 4 M GdnHCl by a reverse thermal gradient (90°C to room temperature over 4 hours). Eluted proteins were subject to size exclusion chromatography (Superdex 75, GE) to separate monomers from aggregates. 5H_LAVA_ contains the affinity-disrupting L556A/V570A mutations in all three N-HR segments.

The concentrations of T20, PIE12, C37 constructs and 5-Helix proteins were determined by absorbance at 280 nm by the method of Edelhoch [83]. The interaction and inhibition properties of these inhibitors have been extensively characterized [50, 55, 66]. Inhibitory titrations were performed using pseudotyped HIV-1 (see above) and either U87.CD4.CXCR4 or HOS-CXCR4+ target cells. Unless otherwise noted, the dependence of infectivity (measured by luciferase activity in target cells) on inhibitor concentration was fit to a Langmuir equation to extract IC_50_ values.

### Crystallization and structure determination

Crystal structures were obtained using NC1 proteins (see above) containing either the double mutation Q552R/L555M or single mutation V549E. These mutations were detected in HIV-1_NL4-3_ propagated in the presence of C37 and C37-KYI. The Q552R and V549E mutations disrupt C-peptide binding affinity approximately 5000-fold and confer resistance to C37 (150-fold) and the higher affinity C37-KYI (6-fold) [55]. The L555M mutation impacts neither the affinity nor potency of C37 or C37-KYI either alone or in combination with Q552R. The mutations were incorporated into the NC1 expression plasmid by Quikchange PCR (Stratagene). Proteins were recombinantly expressed and purified by metal chelate chromatography followed by rp-HPLC (described above). Lyophilized proteins were resuspended in water at a concentration of 12–13 mg/mL and ultracentrifuged at 150,000 x g (30 min, 4°C) to remove particulates.

Crystallization conditions were screened (Index HT and Crystal Screen HT, Hampton Research) by sitting-drop vapor diffusion method using robotically dispensed 100 nL sample volumes (HydraIIPlusOne, Thermo Scientific). Following optimization, diffraction quality crystals with hexagonal prism shape of 150 μm were obtained for NC1_Q552R/L555M_ using 20% w/v PEG 8000, 0.3 M Calcium Acetate, 0.1 M Na Cacodylate, (pH 6.5) and 6.35mg/mL of protein. NC1_V549E_ crystallized into a hexagonal prism of 50 μm in 3 M Sodium Chloride, 0.1 M Sodium Acetate (pH 4.5) and 6 mg/mL of protein. Crystals were frozen in mother liquor supplemented with 27% v/v ethylene glycol and stored in liquid nitrogen until exposed.

Diffraction data sets were collected on beamline's X6A at the National Synchrotron Light Source (NSLS, USA) and 23ID-B at the Advanced Photon Source, Argonne National Laboratory. The images were indexed and integrated using DIALS [84] and scaled using AIMLESS [85]. The structures were solved by molecular replacement using Phaser [86] with the HIV gp41 core structure (PDB ID: 1AIK, [12]) as a search model. The manual rebuilding of the model was performed in Coot [87] and refined in PHENIX ver. 1–10.1–2155 [88]. All crystallographic data are summarized in S2 Table. The structures were validated using ADIT Validation Server as implemented on the RSCB PDB website. Structural illustrations were prepared with PyMOL [89] and Coot.

## Supporting Information

S1 Fig Properties of Env homo- and heterotrimers containing the T20-escape mutation L544S. (A) Infectivity of HIV-1 pseudotyped with Env_HXB2_ trimers (WT or L544S) or Env A/Env B trimers (Ww, Wm, Mw and Mm). Env A and Env B were expressed equally in viral producing cells. Each bar represents the mean ± SEM of three or more independent experiments. A Western blot depicting the expression of gp41 in viral progenitor cell lysates is shown below the graph. (B) Fusion inhibitor titrations of HIV-1 pseudotyped with wild-type Env_HXB2_ (black squares) or the L544S mutant variant (red circles). Titrations were performed with di-C37 (filled symbols, solid lines) and T20 (open symbols, dashed lines). (C-G) Fusion inhibitor titrations of HIV-1 generated from cells expressing EnvA and Env B at ratios of 1:1 (C), 1:9 (D-E) or 9:1 (F-G). Viral populations Ww (black), Wm (red), Mw (green) and Mm (blue) were inhibited using di-C37 (C, E, G) or T20 (D, F). Data points represents the mean ± SEM of at least three independent experiments and have been fit to a simple Langmuir equation (solid lines) to extract IC_50_ values. All infections were performed using U87.CD4.CXCR4 target cells.
(PDF)

S2 Fig Properties of Env homo- and heterotrimers containing the T20-escape mutation V549A. (A) Infectivity of HIV-1 pseudotyped with Env_HXB2_ trimers (WT or V549A) or Env A/Env B trimers (Ww, Wm, Mw and Mm). Env A and Env B were expressed equally in viral producing cells. Each bar represents the mean ± SEM of three or more independent experiments. A Western blot depicting the expression of gp41 in viral progenitor cell lysates is shown below the graph. (B) Fusion inhibitor titrations of HIV-1 pseudotyped with wild-type Env_HXB2_ (black squares) or the V549A mutant variant (red circles). Titrations were performed with C37-KYI (filled symbols, solid lines) and T20 (open symbols, dashed lines). (C-G) Fusion inhibitor titrations of HIV-1 generated from cells expressing EnvA and Env B at ratios of 1:1 (C), 1:9 (D-E) or 9:1 (F-G). Viral populations Ww (black), Wm (red), Mw (green) and Mm (blue) were inhibited using C37-KYI (C, E, G) or T20 (D, F). Data points represents the mean ± SEM of at least three independent experiments and have been fit to a simple Langmuir equation (solid lines) to extract IC_50_ values. All infections were performed using U87.CD4.CXCR4 target cells.
(PDF)

S3 Fig Properties of Env homo- and heterotrimers containing the 5H_LAVA_-escape mutation N656D. (A) Infectivity of HIV-1 pseudotyped with wild type Env_HXB2_ or the N656D variant. Each bar represents the mean ± SEM of three or more independent experiments. The Western blot shows gp41 from lysates of viral progenitor cells expressing either Env_HXB2_ trimers (WT or N656D) or Env A/Env B trimers (Ww, Wm, Mw, Mm). Env A and Env B were expressed at a 1:1 ratio. (B) Fusion inhibitor titrations of HIV-1 pseudotyped with wild-type Env_HXB2_ (black squares) or the N656D mutant variant (red circles). Titrations were performed with 5H_WT_ (filled symbols, solid lines) and 5H_LAVA_ (open symbols, dashed lines). (C-G) Fusion inhibitor titrations of HIV-1 generated from cells expressing EnvA and Env B at ratios of 1:1 (C), 1:9 (D-E) or 9:1 (F-G). Viral populations Ww (black), Wm (red), Mw (green) and Mm (blue) were inhibited using 5H_WT_ (C, E, G) or 5H_LAVA_ (D, F). Data points represents the mean ± SEM of at least three independent experiments and have been fit to a simple Langmuir equation (solid lines) to extract IC_50_ values. Infections of HIV-1 pseudotyped with Env homotrimers were performed using HOS-CXCR4+ cells, while infections of HIV-1 pseudotyped with Env A/Env B mixtures were performed using U87.CD4.CXCR4 target cells.
(PDF)

S4 Fig Structural details of the gp41 trimer-of-hairpins containing a Q552R substitution. (A) Lateral view of the six-helix bundle formed by a 40 amino-acid N-HR segment (N40, grey ribbon) and 37 amino-acid C-HR segment (C37, blue ribbon). The N-terminus of N40 and C-terminus of C37 are found at the top of the structure. The unwound region of the distorted N40 segment is colored orange. Arrows designate the levels of Arg_552_, Met_555_, and Ile_559_ (stick representations) reflecting consecutive *a*-, *d*- and *a*-positions of the canonical 3,4-hydrophobic heptad repeat (see Fig 6A). The orientations of these residues are shown in axial projection in B, C and D, respectively. The meshwork around the amino acids reflects the electron density computed from a (2Fo-Fc) map contoured at 1 sigma. (E) Expanded view of the unwound region of the distorted N-HR helix. The meshwork around the amino acids reflects the electron density computed from a (Fo-Fc) omit-map contoured at 2 sigma. Amino acids shown in stick representation are color coded by atom as follows: carbon—green; nitrogen—blue; oxygen—red; sulfur—yellow.
(PDF)

S5 Fig C-peptide binding sites of the wild type gp41 N-HR coiled coil and the Q552R mutant variant. (Top) Ribbon diagrams depicting structures of the wild type (PDB ID: 1AIK, [12]) and mutant trimer-of-hairpins in axial projection. The N-HR segments are colored gray while the C-HR segments are colored blue. The diagrams are oriented with the N-terminus of the N-HR segment and C-terminus of the C-HR segment coming out of the page. Stick representations of residues 551 (Gln) and 552 (Gln or Arg) are in teal and red, respectively. (Bottom) Expanded view of the C-peptide interface at residue 552. N-HR segments are shown in gray surface rendering except for surface-exposed regions of residues 551 (teal) and 552 (red). The lateral projections are oriented with the N-termini of the N-HR segments on the right. The numbers for the Q552R mutant variant reflect the three different C-peptide binding sites for this N-HR trimer (top). The arrows point to the unwound N-HR segment.
(PDF)

S6 Fig Structural superposition of the trimer-of-hairpins from wild type gp41 (HXB2 sequence; PDB ID: 1AIK [12]) and variants containing the Q552R or V549E substitution. Ribbon diagrams depicting the wild type structure are shown in gray (N-HR segment, 36 amino acids) and blue (C-HR segment, 34 amino acids). (A) The Q552R variant (red) aligns to the wild type structure with a backbone Cα RMSD of 0.69 Å. (B) The V549E variant (green) aligns to the wild type structure with a backbone Cα RMSD of 0.94 Å. The diagrams are oriented with the N-termini of the N-HR segments and C-termini of the C-HR segments at the top of the structures.
(PDF)

S7 Fig Structural details of the gp41 trimer-of-hairpins containing the V549E substitution. (A and C) Ribbon diagrams depicting the structures the wild type trimer-of-hairpins (PDB ID: 1AIK, [12]) and the V549E mutant variant. The N-HR segments are shown in gray, while the C-HR segments are shown in blue. The diagrams are oriented with the N-termini of the N-HR helices on the left. (B and D) Expanded view of the boxed regions in A and C containing residue 549. The N-HR and C-HR regions are modeled in surface representation, and residues 549 (Val or Glu), 656 (Asn), 657 (Glu), and 660 (Leu) are shown in stick representation color coded as follows: carbon-green, nitrogen-blue, oxygen-red.
(PDF)

S8 Fig Asymmetric exposure of the N-HR coiled coil. (Top) Helical wheel diagram of the gp41 TOH showing the relative positions of mutations L544S and V549A on the N-HR coiled coil. For each Env protomer, these residues point into different C-HR binding sites. (Bottom) Modeled exposure of the N-HR coiled coil of A_2_B heterotrimers from Mw viruses. The N-HR helices from the two Env A protomers (gray) contain the L544S (purple asterisk) or V549A (orange asterisk) substitution. The N-HR helix from the Env B protomer (olive) has a wild type sequence. The resulting N-HR coiled coil contains 1 high affinity and 2 low affinity T20 binding sites. A steric barrier (green) blocks C-peptide access to the N-HR coiled coil until the second CD4-Env interaction. If only the site formed by the two Env A protomers is initially exposed, then T20 binds with low affinity regardless of whether the heterotrimer contains the L544S or V549A substitution. However, if two C-peptide binding sites are exposed, then T20 will have access to at least one high affinity site on either the L544S or V549A mutant heterotrimer. As arbitrarily drawn here, barriers covering the *g*-positions of the Env A N-HR helices are removed, allowing T20 access to two low affinity sites for L544S mutant heterotrimers. However, removing the same steric barriers now uncovers one high affinity site on V549A mutant heterotrimers (thick arrow). Under these circumstances, T20 should poorly inhibit L544S mutant heterotrimers but potently inhibit V549A mutant heterotrimers.
(PDF)

S9 Fig Native structures of class 1 viral fusion proteins from influenza and Ebola viruses. (A) Influenza virus hemagglutinin (PDB ID: 3BT6, [90]). (B) Ebola virus GP (PDB ID: 3CSY, [71]). In the orientation depicted, the viral membrane is located below the structures. The surface subunits (HA1 and GP1) are shown in green space-filling representation. The transmembrane subunits (HA2 and GP2) are depicted as ribbon diagrams and color coded as follows: fusion peptide/fusion loop—red; N-terminal heptad repeat (N-HR)—grey; linker region—orange; C-terminal extension that packs against the N-HR coiled coil in the TOH conformation—blue.
(PDF)

S1 Table General properties of fusion inhibitors used in this study. (DOCX)

S2 Table Crystallographic data collection and refinement statistics. (DOCX)
