# Supplementary figures and images for: Receptor Activation of HIV-1 Env Leads to Asymmetric Exposure of the gp41 Trimer
Source: PLoS Pathog. 2016 Dec 19;12(12):e1006098. doi: 10.1371/journal.ppat.1006098 (PMC5222517; doi:10.1371/journal.ppat.1006098)

**A**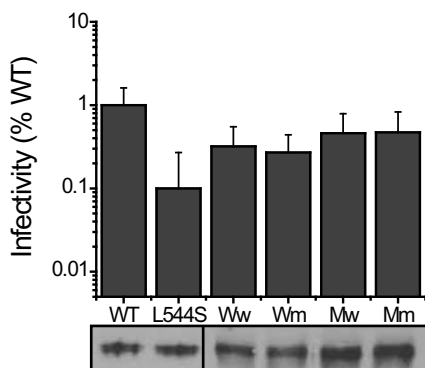

See Fig 4A for T20 titrations  
of Env Heterotrimers

1:1 Expression Ratio (A:B)  
 $A_2B$  and  $AB_2$  Trimers

**B**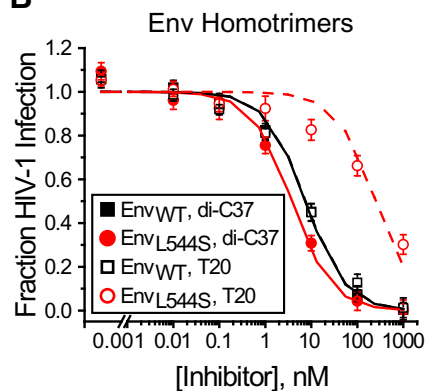**C**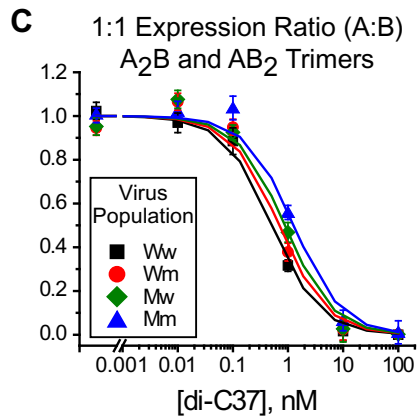**D**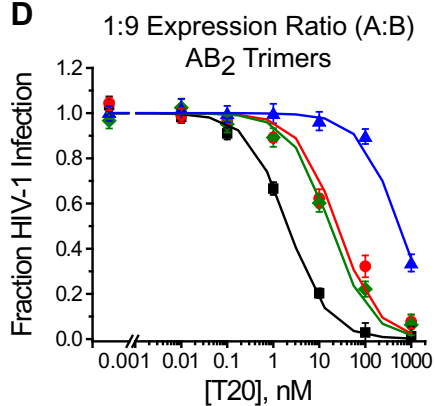**E**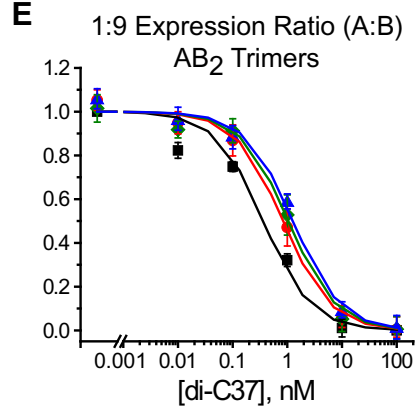**F**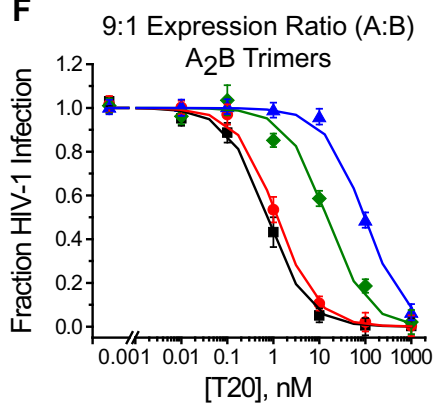**G**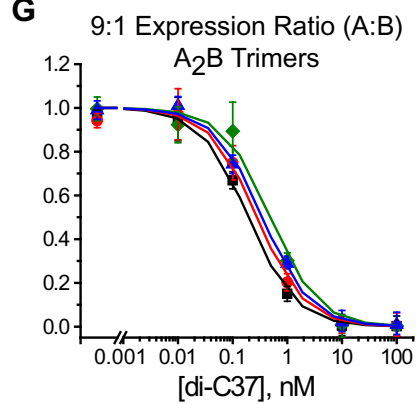

Supplement: S1 Fig — (A) Infectivity of HIV-1 pseudotyped with EnvHXB2 trimers (WT or L544S) or Env A/Env B trimers (Ww, Wm, Mw and Mm). Env A and Env B were expressed equally in viral producing cells. Each bar represents the mean ± SEM of three or more independent experiments. A Western blot depicting the expression of gp41 in viral progenitor cell lysates is shown below the graph. (B) Fusion inhibitor titrations of HIV-1 pseudotyped with wild-type EnvHXB2 (black squares) or the L544S mutant variant (red circles). Titrations were performed with di-C37 (filled symbols, solid lines) and T20 (open symbols, dashed lines). (C-G) Fusion inhibitor titrations of HIV-1 generated from cells expressing EnvA and Env B at ratios of 1:1 (C), 1:9 (D-E) or 9:1 (F-G). Viral populations Ww (black), Wm (red), Mw (green) and Mm (blue) were inhibited using di-C37 (C, E, G) or T20 (D, F). Data points represents the mean ± SEM of at least three independent experiments and have been fit to a simple Langmuir equation (solid lines) to extract IC50 values. All infections were performed using U87.CD4.CXCR4 target cells. (PDF) [file ppat.1006098.s001.pdf]

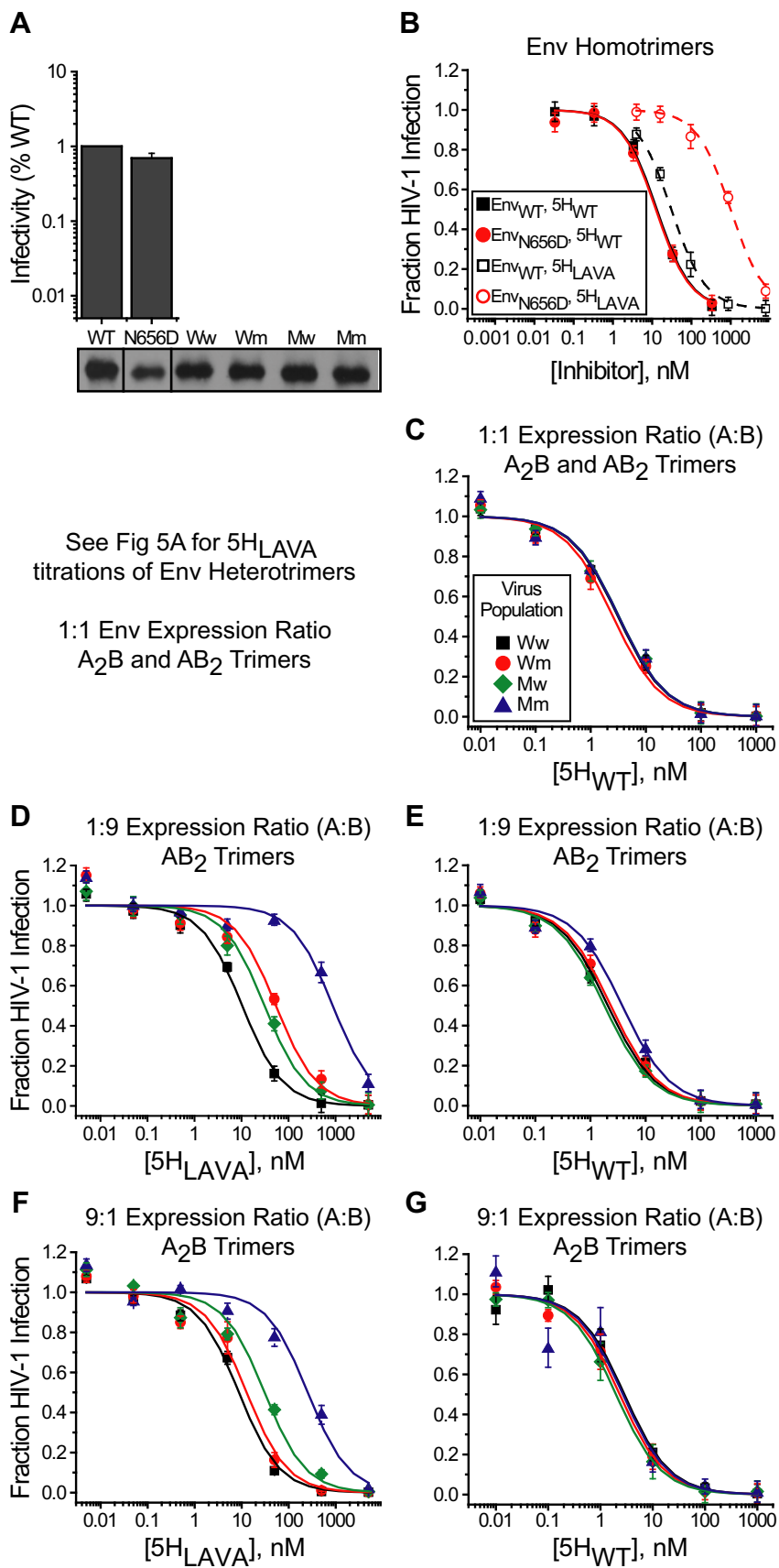

Supplement: S3 Fig — (A) Infectivity of HIV-1 pseudotyped with wild type EnvHXB2 or the N656D variant. Each bar represents the mean ± SEM of three or more independent experiments. The Western blot shows gp41 from lysates of viral progenitor cells expressing either EnvHXB2 trimers (WT or N656D) or Env A/Env B trimers (Ww, Wm, Mw, Mm). Env A and Env B were expressed at a 1:1 ratio. (B) Fusion inhibitor titrations of HIV-1 pseudotyped with wild-type EnvHXB2 (black squares) or the N656D mutant variant (red circles). Titrations were performed with 5HWT (filled symbols, solid lines) and 5HLAVA (open symbols, dashed lines). (C-G) Fusion inhibitor titrations of HIV-1 generated from cells expressing EnvA and Env B at ratios of 1:1 (C), 1:9 (D-E) or 9:1 (F-G). Viral populations Ww (black), Wm (red), Mw (green) and Mm (blue) were inhibited using 5HWT (C, E, G) or 5HLAVA (D, F). Data points represents the mean ± SEM of at least three independent experiments and have been fit to a simple Langmuir equation (solid lines) to extract IC50 values. Infections of HIV-1 pseudotyped with Env homotrimers were performed using HOS-CXCR4+ cells, while infections of HIV-1 pseudotyped with Env A/Env B mixtures were performed using U87.CD4.CXCR4 target cells. (PDF) [file ppat.1006098.s003.pdf]

A

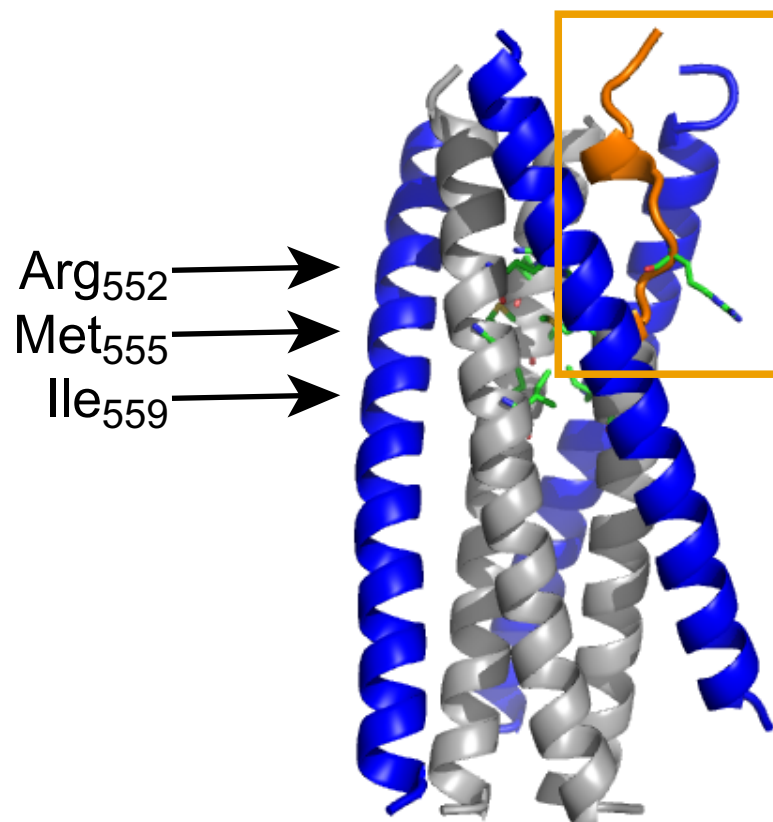

E

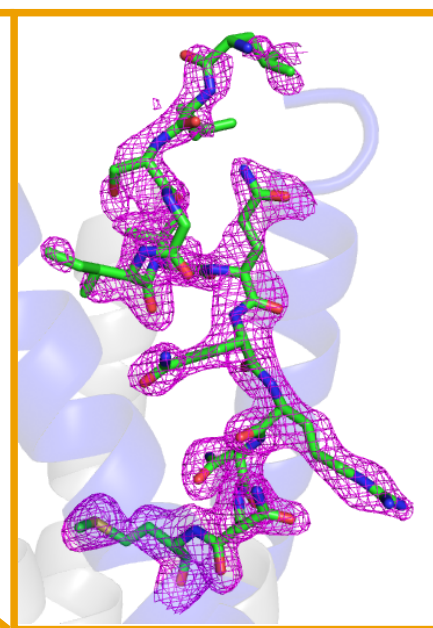

B

Arg<sub>552</sub>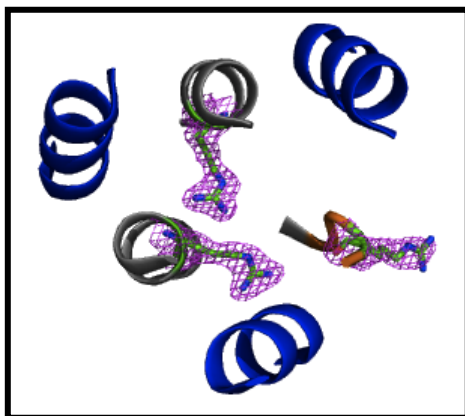

C

Met<sub>555</sub>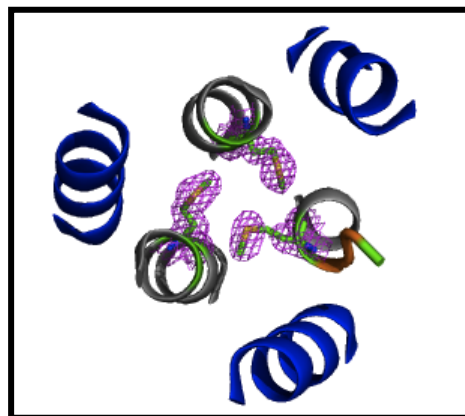

D

Ile<sub>559</sub>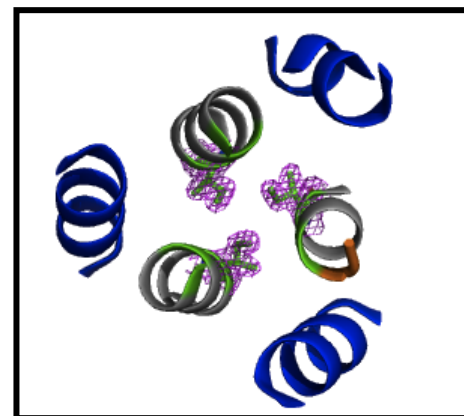

Supplement: S4 Fig — (A) Lateral view of the six-helix bundle formed by a 40 amino-acid N-HR segment (N40, grey ribbon) and 37 amino-acid C-HR segment (C37, blue ribbon). The N-terminus of N40 and C-terminus of C37 are found at the top of the structure. The unwound region of the distorted N40 segment is colored orange. Arrows designate the levels of Arg552, Met555, and Ile559 (stick representations) reflecting consecutive a-, d- and a-positions of the canonical 3,4-hydrophobic heptad repeat (see Fig 6A). The orientations of these residues are shown in axial projection in B, C and D, respectively. The meshwork around the amino acids reflects the electron density computed from a (2Fo-Fc) map contoured at 1 sigma. (E) Expanded view of the unwound region of the distorted N-HR helix. The meshwork around the amino acids reflects the electron density computed from a (Fo-Fc) omit-map contoured at 2 sigma. Amino acids shown in stick representation are color coded by atom as follows: carbon—green; nitrogen—blue; oxygen—red; sulfur—yellow. (PDF) [file ppat.1006098.s004.pdf]

Wild Type

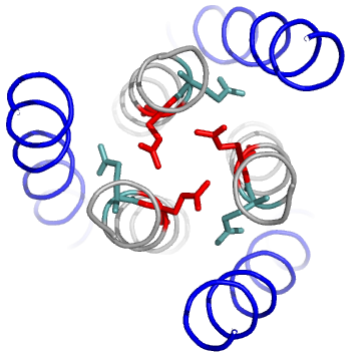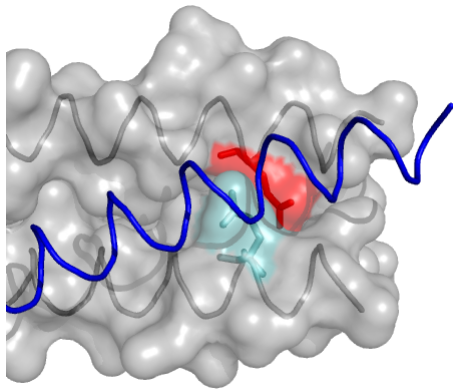

Gln<sub>551</sub>  
Gln<sub>552</sub> or Arg<sub>552</sub>

Q552R

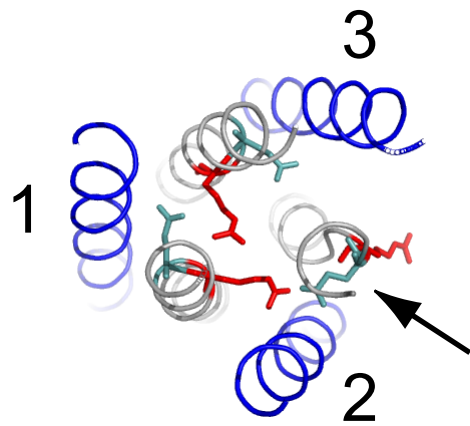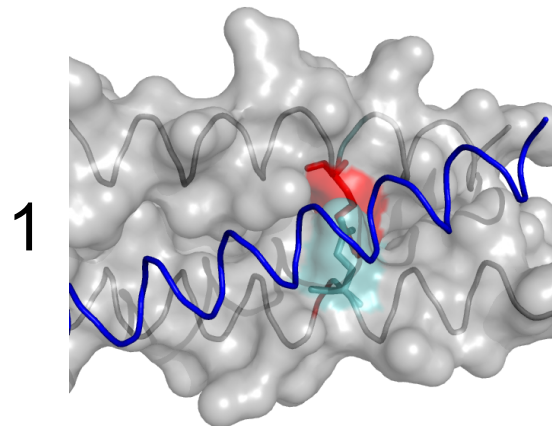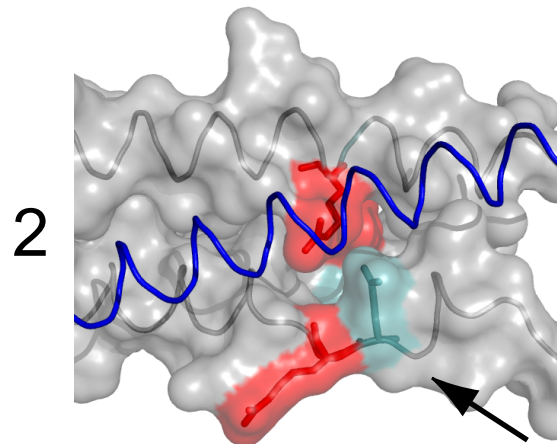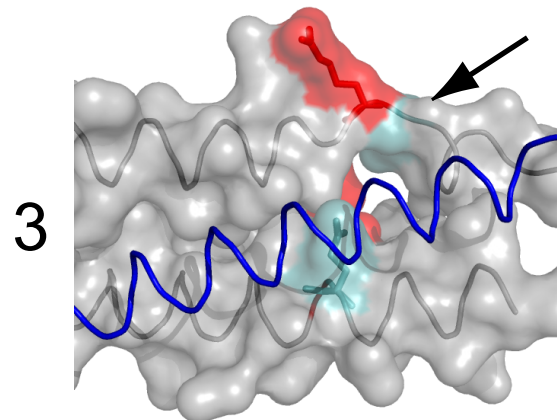

Supplement: S5 Fig — (Top) Ribbon diagrams depicting structures of the wild type (PDB ID: 1AIK, [12]) and mutant trimer-of-hairpins in axial projection. The N-HR segments are colored gray while the C-HR segments are colored blue. The diagrams are oriented with the N-terminus of the N-HR segment and C-terminus of the C-HR segment coming out of the page. Stick representations of residues 551 (Gln) and 552 (Gln or Arg) are in teal and red, respectively. (Bottom) Expanded view of the C-peptide interface at residue 552. N-HR segments are shown in gray surface rendering except for surface-exposed regions of residues 551 (teal) and 552 (red). The lateral projections are oriented with the N-termini of the N-HR segments on the right. The numbers for the Q552R mutant variant reflect the three different C-peptide binding sites for this N-HR trimer (top). The arrows point to the unwound N-HR segment. (PDF) [file ppat.1006098.s005.pdf]

A

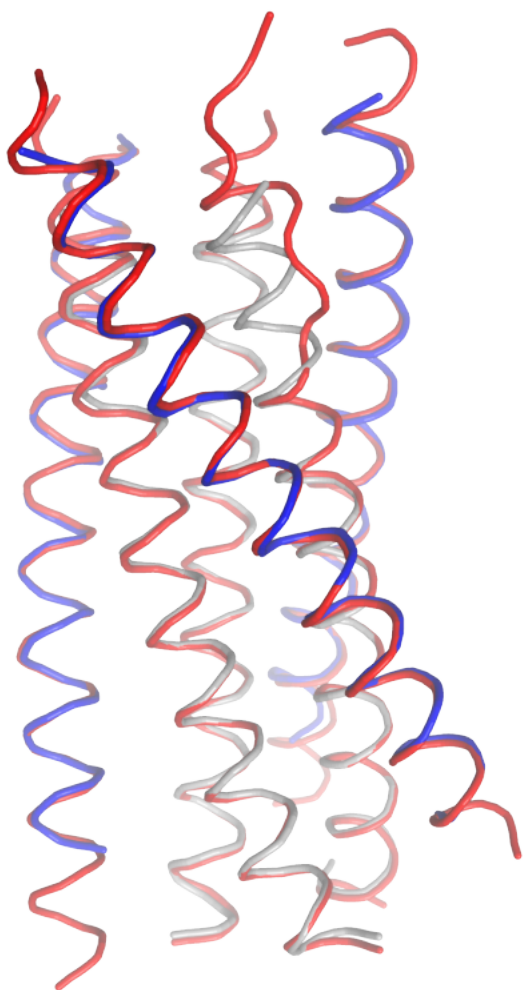

B

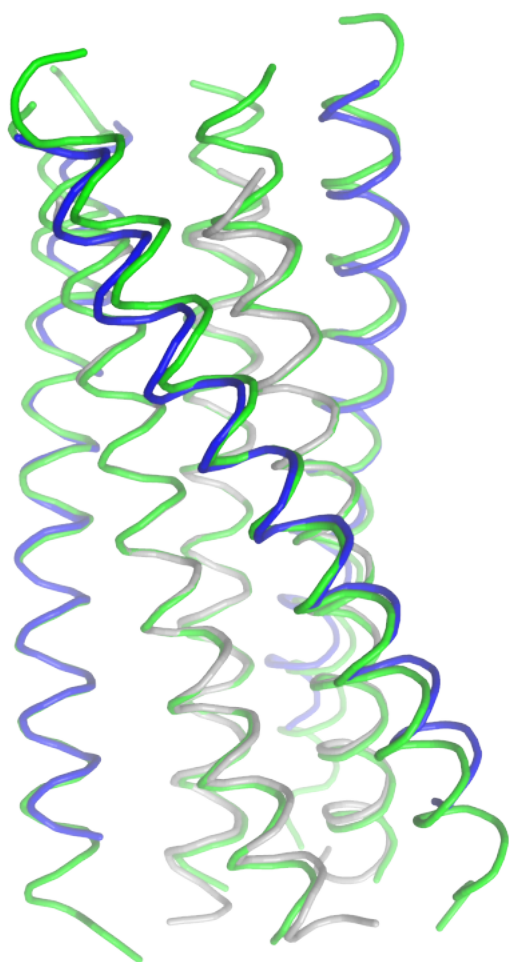

Supplement: S6 Fig — Ribbon diagrams depicting the wild type structure are shown in gray (N-HR segment, 36 amino acids) and blue (C-HR segment, 34 amino acids). (A) The Q552R variant (red) aligns to the wild type structure with a backbone Cα RMSD of 0.69 Å. (B) The V549E variant (green) aligns to the wild type structure with a backbone Cα RMSD of 0.94 Å. The diagrams are oriented with the N-termini of the N-HR segments and C-termini of the C-HR segments at the top of the structures. (PDF) [file ppat.1006098.s006.pdf]

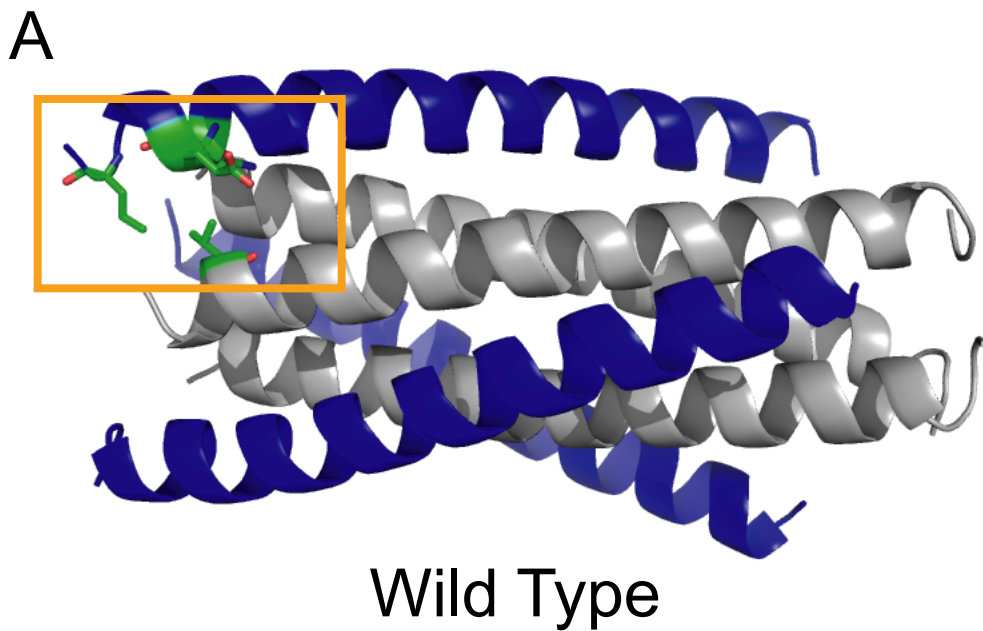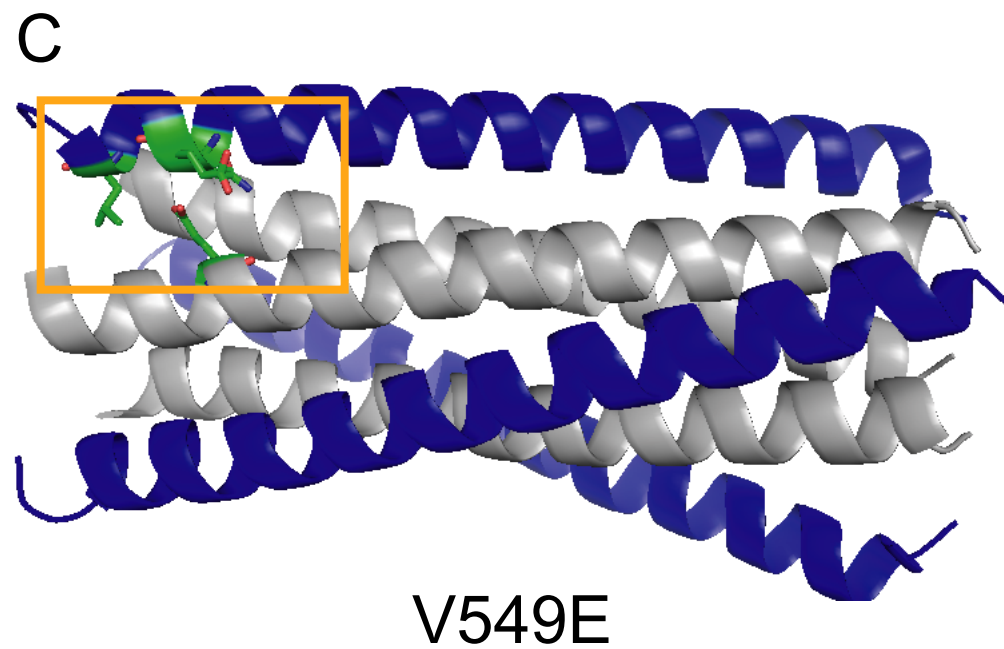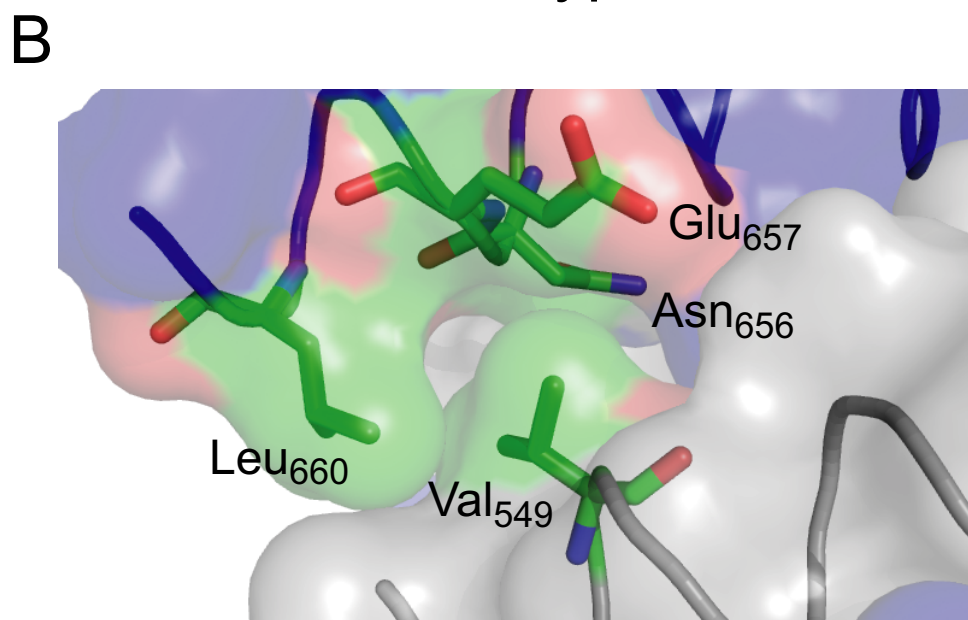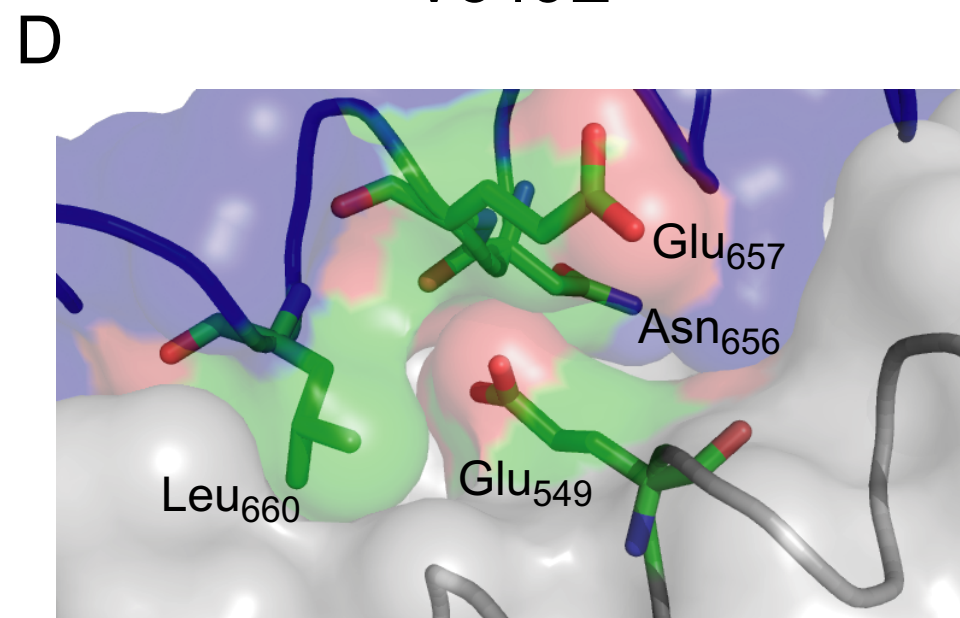

Supplement: S7 Fig — (A and C) Ribbon diagrams depicting the structures the wild type trimer-of-hairpins (PDB ID: 1AIK, [12]) and the V549E mutant variant. The N-HR segments are shown in gray, while the C-HR segments are shown in blue. The diagrams are oriented with the N-termini of the N-HR helices on the left. (B and D) Expanded view of the boxed regions in A and C containing residue 549. The N-HR and C-HR regions are modeled in surface representation, and residues 549 (Val or Glu), 656 (Asn), 657 (Glu), and 660 (Leu) are shown in stick representation color coded as follows: carbon-green, nitrogen-blue, oxygen-red. (PDF) [file ppat.1006098.s007.pdf]

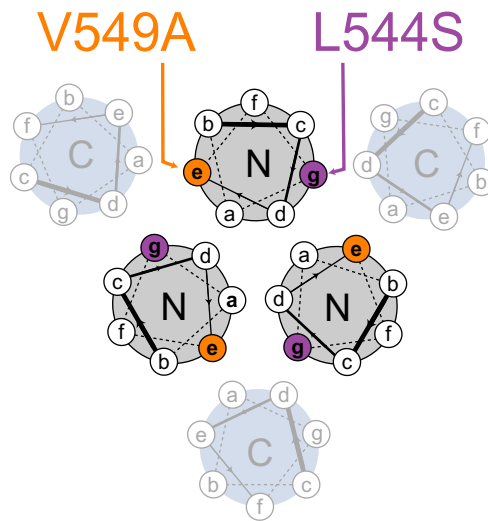

1 CD4 Bound

No Sites  
Exposed

2 CD4 Bound

1 Site  
Exposed

2 Sites  
Exposed

L544S

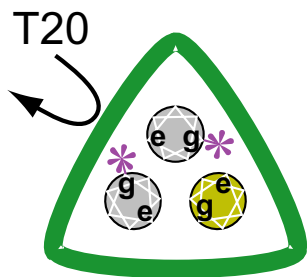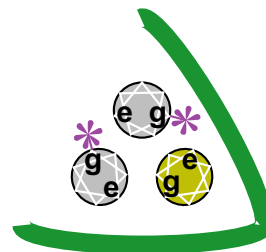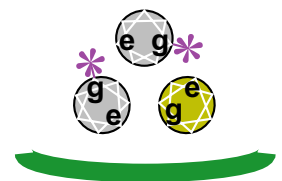

V529A

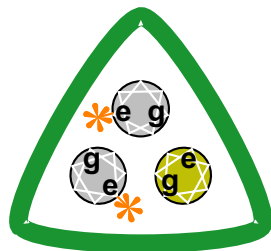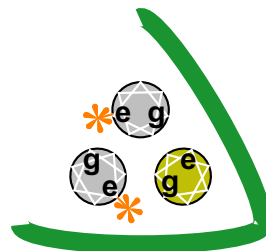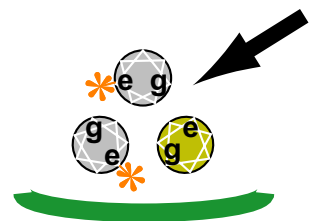

Supplement: S8 Fig — (Top) Helical wheel diagram of the gp41 TOH showing the relative positions of mutations L544S and V549A on the N-HR coiled coil. For each Env protomer, these residues point into different C-HR binding sites. (Bottom) Modeled exposure of the N-HR coiled coil of A2B heterotrimers from Mw viruses. The N-HR helices from the two Env A protomers (gray) contain the L544S (purple asterisk) or V549A (orange asterisk) substitution. The N-HR helix from the Env B protomer (olive) has a wild type sequence. The resulting N-HR coiled coil contains 1 high affinity and 2 low affinity T20 binding sites. A steric barrier (green) blocks C-peptide access to the N-HR coiled coil until the second CD4-Env interaction. If only the site formed by the two Env A protomers is initially exposed, then T20 binds with low affinity regardless of whether the heterotrimer contains the L544S or V549A substitution. However, if two C-peptide binding sites are exposed, then T20 will have access to at least one high affinity site on either the L544S or V549A mutant heterotrimer. As arbitrarily drawn here, barriers covering the g-positions of the Env A N-HR helices are removed, allowing T20 access to two low affinity sites for L544S mutant heterotrimers. However, removing the same steric barriers now uncovers one high affinity site on V549A mutant heterotrimers (thick arrow). Under these circumstances, T20 should poorly inhibit L544S mutant heterotrimers but potently inhibit V549A mutant heterotrimers. (PDF) [file ppat.1006098.s008.pdf]

**A**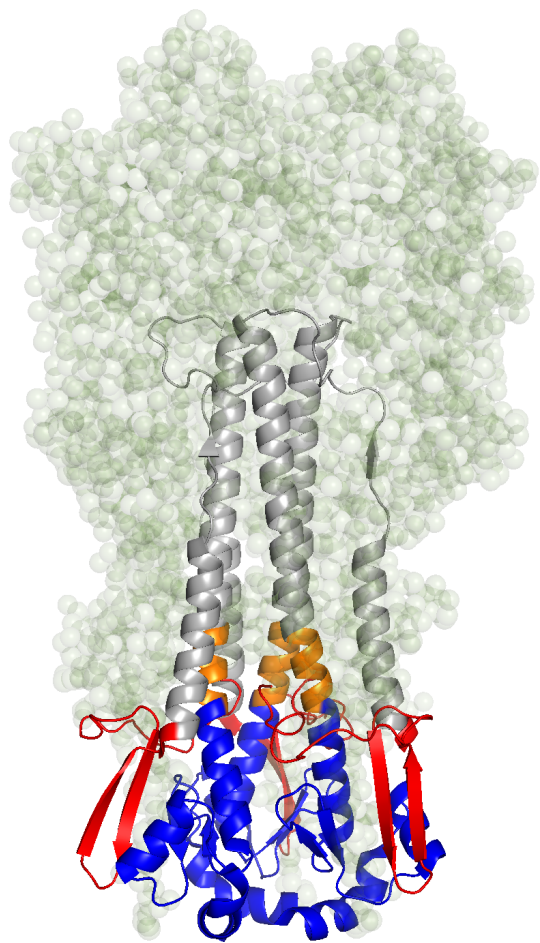**B**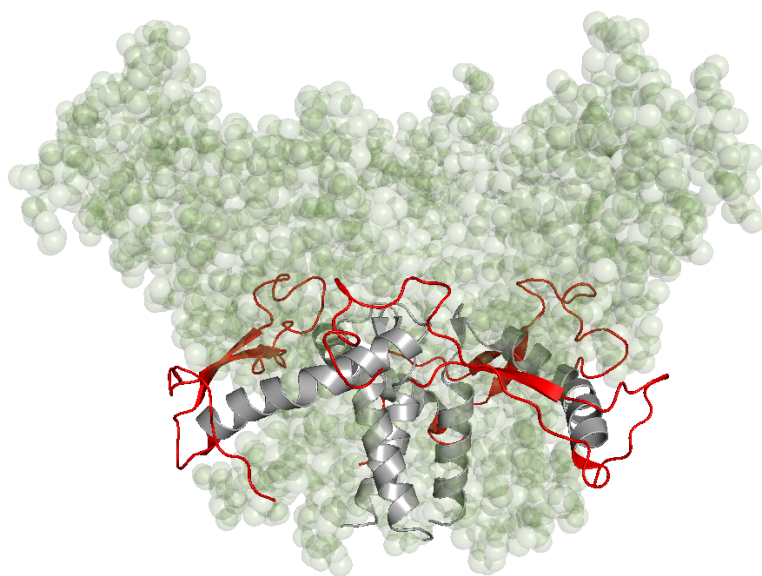

Supplement: S9 Fig — (A) Influenza virus hemagglutinin (PDB ID: 3BT6, [90]). (B) Ebola virus GP (PDB ID: 3CSY, [71]). In the orientation depicted, the viral membrane is located below the structures. The surface subunits (HA1 and GP1) are shown in green space-filling representation. The transmembrane subunits (HA2 and GP2) are depicted as ribbon diagrams and color coded as follows: fusion peptide/fusion loop—red; N-terminal heptad repeat (N-HR)—grey; linker region—orange; C-terminal extension that packs against the N-HR coiled coil in the TOH conformation—blue. (PDF) [file ppat.1006098.s009.pdf]
