# Supplementary material for: Receptor Activation of HIV-1 Env Leads to Asymmetric Exposure of the gp41 Trimer
Source: PLoS Pathog. 2016 Dec 19;12(12):e1006098. doi: 10.1371/journal.ppat.1006098 (PMC5222517; doi:10.1371/journal.ppat.1006098)
Supplement: S2 Fig — (A) Infectivity of HIV-1 pseudotyped with EnvHXB2 trimers (WT or V549A) or Env A/Env B trimers (Ww, Wm, Mw and Mm). Env A and Env B were expressed equally in viral producing cells. Each bar represents the mean ± SEM of three or more independent experiments. A Western blot depicting the expression of gp41 in viral progenitor cell lysates is shown below the graph. (B) Fusion inhibitor titrations of HIV-1 pseudotyped with wild-type EnvHXB2 (black squares) or the V549A mutant variant (red circles). Titrations were performed with C37-KYI (filled symbols, solid lines) and T20 (open symbols, dashed lines). (C-G) Fusion inhibitor titrations of HIV-1 generated from cells expressing EnvA and Env B at ratios of 1:1 (C), 1:9 (D-E) or 9:1 (F-G). Viral populations Ww (black), Wm (red), Mw (green) and Mm (blue) were inhibited using C37-KYI (C, E, G) or T20 (D, F). Data points represents the mean ± SEM of at least three independent experiments and have been fit to a simple Langmuir equation (solid lines) to extract IC50 values. All infections were performed using U87.CD4.CXCR4 target cells. (PDF) [file ppat.1006098.s002.pdf]

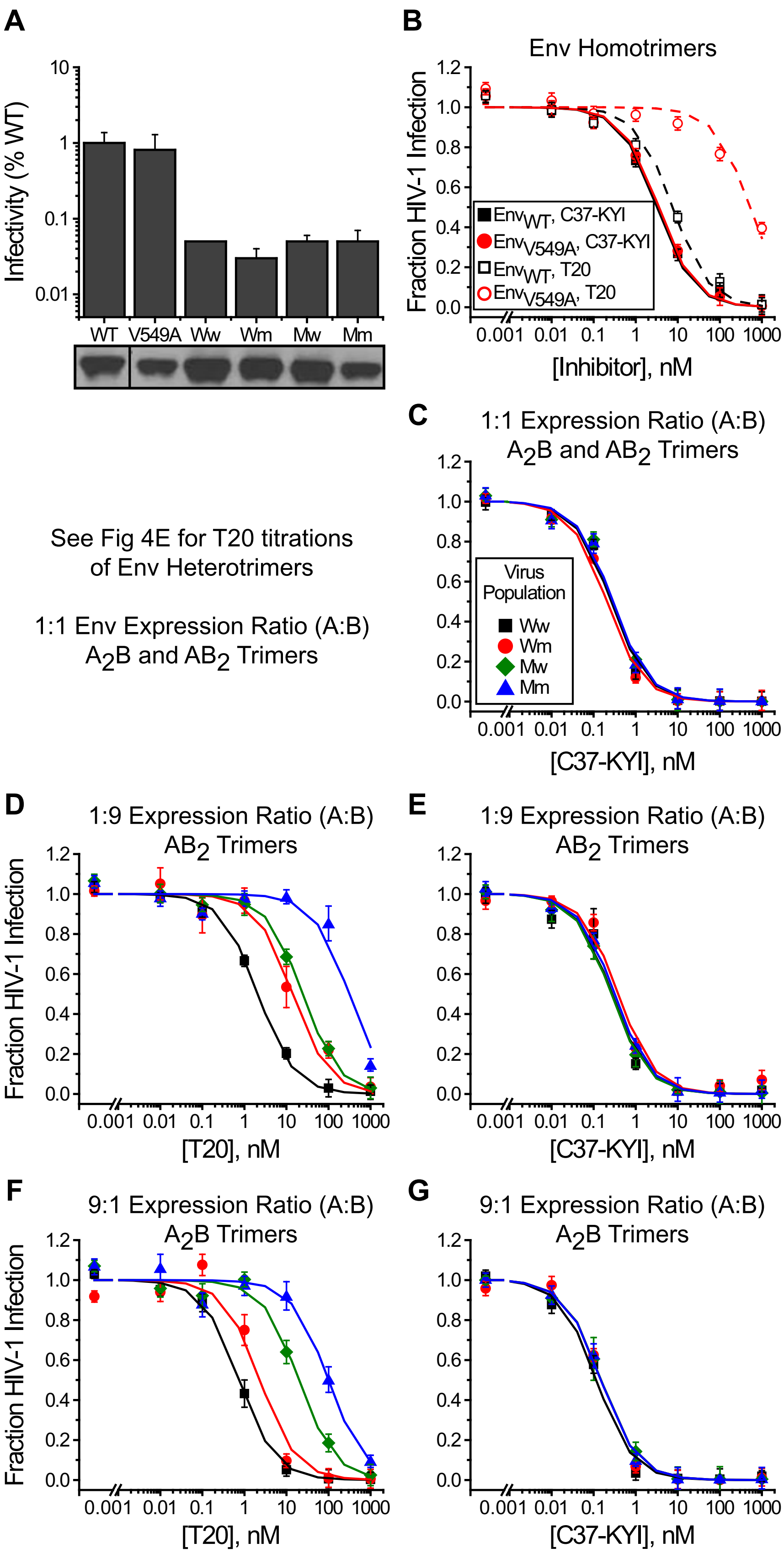
A
Infectivity (% WT)
10
1
0.1
0.01
WT
V549A
Ww
Wm
Mw
Mm
B
Env Homotrimers
Fraction HIV-1 Infection
Env$_{WT}$, C37-KYI
Env$_{V549A}$, C37-KYI
Env$_{WT}$, T20
Env$_{V549A}$, T20
[Inhibitor], nM
0.001 0.01 0.1 1 10 100 1000
See Fig 4E for T20 titrations of Env Heterotrimers
1:1 Env Expression Ratio (A:B)
$A_2B$ and $AB_2$ Trimers
C
1:1 Expression Ratio (A:B)
$A_2B$ and $AB_2$ Trimers
Virus Population
Ww
Wm
Mw
Mm
[C37-KYI], nM
D
1:9 Expression Ratio (A:B)
$AB_2$ Trimers
Fraction HIV-1 Infection
[T20], nM
E
1:9 Expression Ratio (A:B)
$AB_2$ Trimers
[C37-KYI], nM
F
9:1 Expression Ratio (A:B)
$A_2B$ Trimers
Fraction HIV-1 Infection
[T20], nM
G
9:1 Expression Ratio (A:B)
$A_2B$ Trimers
[C37-KYI], nM
