# Supplementary material for: Receptor Activation of HIV-1 Env Leads to Asymmetric Exposure of the gp41 Trimer
Source: PLoS Pathog. 2016 Dec 19;12(12):e1006098. doi: 10.1371/journal.ppat.1006098 (PMC5222517; doi:10.1371/journal.ppat.1006098)
Supplement: S1 Table — (DOCX) [file ppat.1006098.s010.docx]

**Table S1: General properties of fusion inhibitors used in this study**

| Inhibitor | Binding site on gp41 | Env variant | Inhibitor Classification* |
| --- | --- | --- | --- |
| T20^1^ | N-HR | WT | AD |
|  |  | L544S | AD |
|  |  | V549A | AD |
|  |  |  |  |
| di-C37^2^ | N-HR | WT | KR |
|  |  | L544S | KR |
|  |  | V549E/N637K | KR |
|  |  | Q552R/N637K | KR |
|  |  |  |  |
| C37-KYI^2^ | N-HR | WT | KR |
|  |  | V549A | KR |
|  |  | V549E/N637K | AD |
|  |  | Q552R/N637K | AD |
|  |  |  |  |
| PIE12^3^ | N-HR pocket | WT | AD |
|  |  | V549E/N637K | AD |
|  |  | Q552R/N637K | AD |
|  |  |  |  |
| 5H_WT_^2.4^ | C-HR | WT | KR |
|  |  | N656D | KR |
|  |  | V549E/N637K | KR |
|  |  | Q552R/N637K | KR |
|  |  |  |  |
| 5H_LAVA_^2,4^ | C-HR | WT | AD |
|  |  | N656D | AD |

*AD – affinity dependent; KR – kinetically restricted

1. Wild CT, Shugars DC, Greenwell TK, McDanal CB, Matthews TJ. Peptides corresponding to a predictive alpha-helical domain of human immunodeficiency virus type 1 gp41 are potent inhibitors of virus infection. Proc Natl Acad Sci U S A. 1994 Oct 11;91(21):9770-4.
2. Kahle KM, Steger HK, Root MJ. Asymmetric deactivation of HIV-1 gp41 following fusion inhibitor binding. PLoS Pathog. 2009 Nov;5(11):e1000674.
3. Welch BD, Francis JN, Redman JS, Paul S, Weinstock MT, Reeves JD, et al. Design of a potent D-peptide HIV-1 entry inhibitor with a strong barrier to resistance. J Virol. 2010 Nov;84(21):11235-44.
4. Steger HK, Root MJ. Kinetic dependence to HIV-1 entry inhibition. J Biol Chem. 2006 Sep 1;281(35):25813-21.
