# Supplementary material for: Receptor Activation of HIV-1 Env Leads to Asymmetric Exposure of the gp41 Trimer
Source: PLoS Pathog. 2016 Dec 19;12(12):e1006098. doi: 10.1371/journal.ppat.1006098 (PMC5222517; doi:10.1371/journal.ppat.1006098)
Supplement: S2 Table — (DOCX) [file ppat.1006098.s011.docx]

**S2 Table. Crystallographic data collection and refinement statistics.**

|  | **Q552R L555M gp41** | **V549E gp41** |
| --- | --- | --- |
| **Data collection statistics** |  |  |
| X-ray source | NSLS beamline X6A | APS beamline 23-ID-B |
| X-ray detector | ADSC Q270 CCD | MARMosaic 300 CCD |
| Wavelength (Å) | 0.98 | 1.0 |
| Space group | *P*3_1_ | *R*3 :H |
| Resolution (Å) | 36.00 - 1.85 (1.85 - 1.90) *^a^* | 18.00 – 1.8 (1.85 - 1.80) |
| Unit cell dimensions (Å) | *a*, *b* = 43.59, *c* = 113.05 | *a*, *b* = 40.72, *c* = 115.52 |
| Angles (°) | α, β = 90, γ = 120 | α, β = 90 γ = 120 |
| Solvent content (%) / *V*_M_ (Å^3^/Da.) | 42.6 / 2.01 | 31.7 / 1.8 |
| Molecules per asymmetric unit | 3 | 1 |
| Total reflections | 131,722 | 18,638 |
| Unique reflections | 20,547 (1567) | 6619 (497) |
| Multiplicity | 6.4 (6.4) | 2.8 (2.8) |
| Completeness (%) | 99.9 (100.0) | 99.8 (99.5) |
| Mean I/sigma(I) | 11.9 (1.2) | 6.3 (1.3) |
| Wilson B-factor (Å^2^) | 29.26 | 24.25 |
| *R*_merge_ *^b^* | 0.07 (1.623) | 0.064 (0.597) |
| *R*_meas_ *^c^* | 0.077 (1.767) | 0.079 (0.747) |
| *R*_pim_ *^d^* | 0.030 (0.697) | 0.046 (0.445) |
| CC_1/2_ *^e^* | 0.999 (0.668) | 0.997 (0.658) |
| **Refinement statistics** |  |  |
| Resolution (Å) | 31.4 - 1.85 (1.92 - 1.85) | 18.0 – 1.80 (1.864 – 1.8) |
| Number of Reflections | 129,081 (11,756) | 18,634 (1833) |
| Reflections used for *R*_free_ *^f^* | 1961 (190) | 669 (68) |
| CC* *^e^* | 1.0 (0.785) | 0.999 (0.703) |
| *R*_work_ | 0.2309 | 0.1871 |
| *R*_free_ *^f^* | 0.2776 | 0.2225 |
| CC_work_ *^e^* | 0.962 (0.537) | 0.962 (0.565) |
| CC_free_ *^e^* | 0.955 (0.468) | 0.907 (0.652) |
| Twin law | - | Merohedral -h-k,k,-l |
| Number of non-hydrogen atoms | 2035 | 679 |
| - macromolecules | 1939 | 647 |
| - water | 96 | 32 |
| Protein residues | 235 | 79 |
| **Root mean square deviations from ideal geometry** |  |  |
| bond length (Å) | 0.003 | 0.002 |
| angles (°) | 0.532 | 0.321 |
| **Ramachandran plot and MolProbity validation** *^g^* |  |  |
| Residues in favored region (%) | 100 | 98.7 |
| Residues in allowed region (%) | 0 | 1.3 |
| Residues as outliers (%) | 0 | 0 |
| Clashscore | 7.25 | 2.35 |
| Overall score | 1.4 | 1.02 |
| **Average B-factor (Å^2^)** |  |  |
| Model (all atoms) | 44.26 | 34.52 |
| Protein | 43.98 | 34.10 |
| Water | 49.85 | 43.15 |
| Number of TLS groups | 7 | 2 |
| **PDB code** | **5KA6** | **5KA5** |

*^a^* Statistics for the highest-resolution shell are shown in parentheses.

*^b^* The simple merging *R* factor for the multiple observations [1, 2].

*^c^* Redundancy-independent merging *R* factor [3].

*^d^* Precision-indicating merging *R* factor [4].

*^e^* The CC_1/2_ is the correlation coefficient between two randomly selected half-datasets; CC* is a statistic metric for assessing the effective resolution limits of data and quality of unmerged data in the context of a refined model; CC_work_ and CC_free_ are the standard and cross-validated correlations of the observed intensities to the refined model-based intensities, for the work and test sets respectively [5].

*^f^* *R*_free_ value is calculated using the small subset of randomly selected reflections (test-set) that are set aside prior to refinement and not used in the refinement of the structural model [6].

*^g^* From [7].

**References**

1. Arndt UW, Crowther RA, Mallett JF. A computer-linked cathode-ray tube microdensitometer for x-ray crystallography. J Sci Instrum. 1968 May;1(5):510-6.

2. Blundell TL, Johnson LN. Protein Crystallography. London: Academic Press; 1976.

3. Diederichs K, Karplus PA. Improved R-factors for diffraction data analysis in macromolecular crystallography. Nat Struct Biol. 1997 Apr;4(4):269-75.

4. Weiss MS. Global indicators of X-ray data quality. J Appl Crystallogr. 2004;34:130-5.

5. Karplus PA, Diederichs K. Linking crystallographic model and data quality. Science. 2012 May 25;336(6084):1030-3.

6. Brunger AT. Free R value: a novel statistical quantity for assessing the accuracy of crystal structures. Nature. 1992 Jan 30;355(6359):472-5.

7. Chen VB, Arendall WB, 3rd, Headd JJ, Keedy DA, Immormino RM, Kapral GJ, et al. MolProbity: all-atom structure validation for macromolecular crystallography. Acta Crystallogr D Biol Crystallogr. 2010 Jan;66(Pt 1):12-21.
